# Protein nanoparticle-induced osmotic pressure gradients modify pulmonary edema through hyperpermeability in acute respiratory distress syndrome

**DOI:** 10.1186/s12951-022-01519-1

**Published:** 2022-07-06

**Authors:** ZhiZhi Qian, QianYi Wang, ZhaoShun Qiu, DanYang Li, ChenCheng Zhang, XiYu Xiong, ZiHui Zheng, QinLi Ruan, YiChen Guo, Jun Guo

**Affiliations:** 1 School of Medicine & Holistic Integrative Medicine, Nanjing University of Chinese Medicine, Nanjing, 210023 Jiangsu People’s Republic of China; 2 Key Laboratory of Drug Target and Drug for Degenerative Disease, Nanjing University of Chinese Medicine, Nanjing, 210023 Jiangsu People’s Republic of China; 3 Biomedical Engineering, University of Alabama at Birmingham School of Medicine, Birmingham, AL USA

**Keywords:** ARDS, Pulmonary edema, Protein nanoparticle-induced osmotic pressure, Voltage-dependent ion channels, Multi-targeted blockade

## Abstract

Acute respiratory distress syndrome (ARDS), caused by noncardiogenic pulmonary edema (PE), contributes significantly to Coronavirus 2019 (COVID-19)-associated morbidity and mortality. We explored the effect of transmembrane osmotic pressure (OP) gradients in PE using a fluorescence resonance energy transfer-based Intermediate filament (IF) tension optical probe. Angiotensin-II- and bradykinin-induced increases in intracellular protein nanoparticle (PN)-OP were associated with inflammasome production and cytoskeletal depolymerization. Intracellular protein nanoparticle production also resulted in cytomembrane hyperpolarization and L-VGCC-induced calcium signals, which differed from diacylglycerol-induced calcium increment via TRPC6 activation. Both pathways involve voltage-dependent cation influx and OP upregulation via SUR1-TRPM4 channels. Meanwhile, intra/extracellular PN-induced OP gradients across membranes upregulated pulmonary endothelial and alveolar barrier permeability. Attenuation of intracellular PN, calcium signals, and cation influx by drug combinations effectively relieved intracellular OP and pulmonary endothelial nonselective permeability, and improved epithelial fluid absorption and PE. Thus, PN-OP is pivotal in pulmonary edema in ARDS and COVID-19, and transmembrane OP recovery could be used to treat pulmonary edema and develop new drug targets in pulmonary injury.

**Graphical Abstract:**

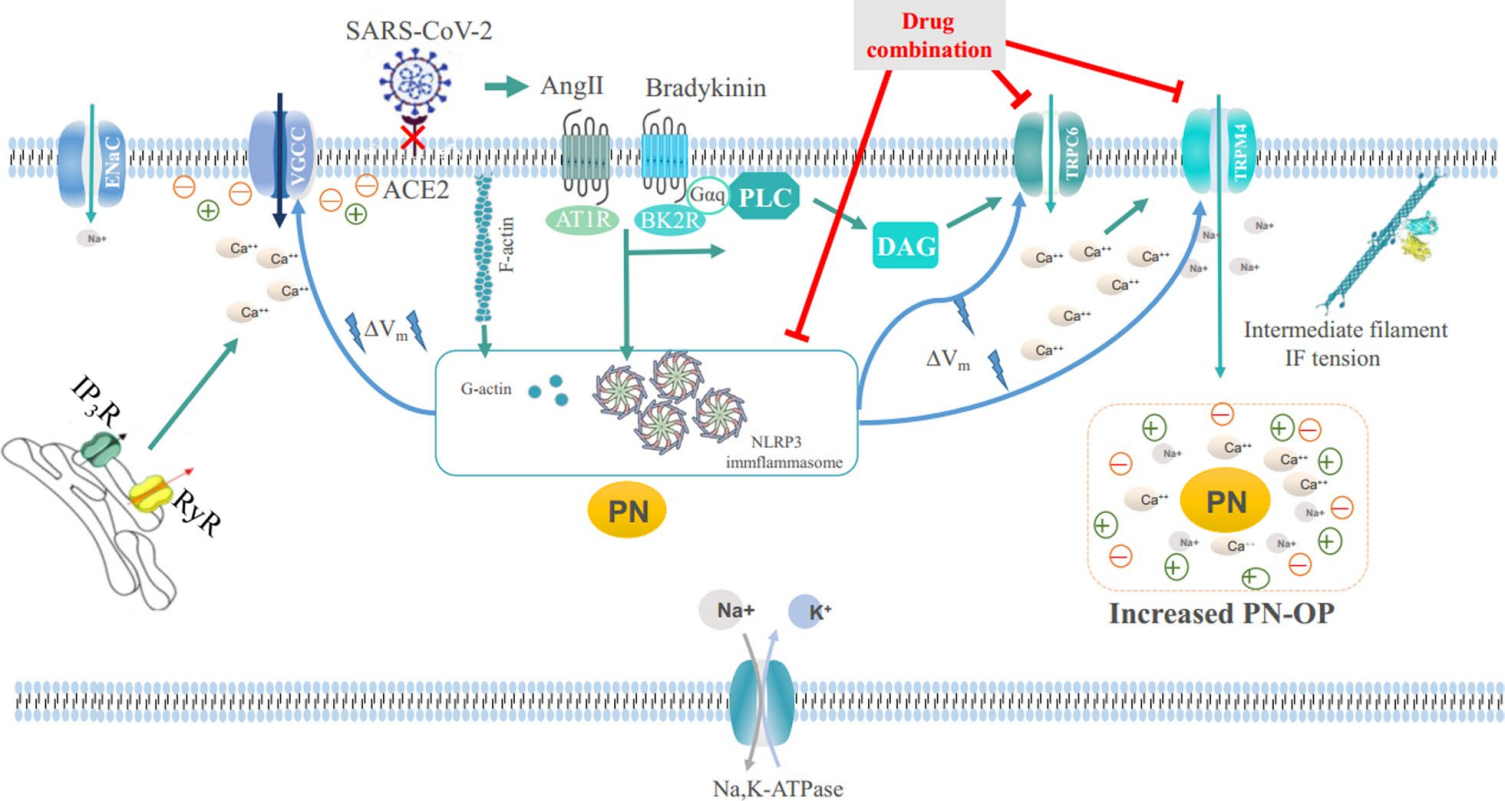

**Supplementary Information:**

The online version contains supplementary material available at 10.1186/s12951-022-01519-1.

## Introduction

Acute respiratory distress syndrome (ARDS) is an acute respiratory illness, caused by alveolar injury secondary to inflammation, either pulmonary or systemic in origin [1]. ARDS is characterized by an inflammatory reaction, increased vascular permeability, and non-cardiogenic pulmonary edema [2]. Based on recent research, ARDS occurred in 41.8% of patients with Coronavirus 2019 (COVID-19), and almost all patients who died from COVID-19 suffered from ARDS [3, 4]. However, in ARDS, pulmonary edema fluid accumulates in the interstitium and air spaces of the lungs, causing increased breathing strain and poor gas exchange, leading to hypoxemia, reduced carbon dioxide excretion, and ultimately, acute respiratory failure [5]. Currently, there is no effective pharmacotherapy for this condition; therefore, it is critical to further study the pathogenesis of ARDS to develop novel targeted therapies.

A previous study suggested that the SARS-CoV2 spike protein on the viral envelope binds to its receptor, angiotensin-converting enzyme 2 (ACE2), causes downregulation of its activity on the target cell, which is involved in lung physiology and pathology through Renin–Angiotensin (RAS) and Kinin–Kallikrein (KKS) systems [6]. The downregulation of ACE2 activity decreases its ability to cleave angiotensin I (AngI) and angiotensin II (AngII) to Ang-(1–9) and Ang-(1–7), respectively, leading to an increased level of AngII and hyperactivation of the angiotensin II type 1 (AT1) receptor [7, 8]. AngII–AT1R axis that regulates vasoconstrictive, proinflammatory, profibrotic, and prooxidative effects is a potential mechanism of cytokine storm and an acute respiratory distress syndrome (ARDS) [9]. Meanwhile, the downregulation of ACE2 activity increases the levels of bradykinin [10]. The binding of bradykinin and the bradykinin receptor 2 (BK2) causes the fluid extravasation and recruitment of leukocytes to the lung, which increase levels of inflammatory mediators through the bradykinin system, resulting in vascular permeability increase and ARDS [11]. ARDS is accompanied by alveolar hyperpermeability, leading to the accumulation of proinflammatory factors and the generation of inflammatory responses [12]. However, how the spike protein, AngII, and BK induce pulmonary edema via the inflammatory response is still uncertain.

Water metabolism and edema occurrence depend on transmembrane oncotic pressure gradients in the body [13]. Based on a recent study, protein nanoparticle production can result in increased osmotic pressure (PN-OP) and water flow, which is also thought to involve upregulation of membrane fluidity and promotes nonselective permeability [14, 15]. Previous studies suggested that ARDS is associated with the secretion of proinflammatory cytokines and inflammasome production [1, 16, 17]. However, more research is needed to verify the relationship between PN-OP, inflammasomes, and pulmonary edema.

The intracellular Ca^2+^ concentration ([Ca^2+^] i) increases in a variety of cells after cell swelling [18, 19]. In pulmonary edema, Ca^2+^ is also an important signaling molecule in alveolar epithelial cells (AECs) [20]. Changes in [Ca^2+^] i have distinct physiological effects on AECs, including membrane reorganization, secretion of surfactants, regulation of channels, and repair of the epithelial barrier [21, 22]. Meanwhile, [Ca^2+^] i has a significant role in NLR family pyrin domain containing 3 (NLRP3) inflammasome activation [23]. Generally, the endoplasmic reticulum is the major source of Ca^2+^ in various cells [24, 25]. Moreover, accumulating evidence shows that transient receptor potential (TRP) channels, which have a high permeability to Ca^2+^ in most sub-families, are involved in lung pathophysiology (e.g., chronic inflammation, fibrosis, and edema) [26]. Numerous reports showed that several TRP channels (such as TRPM2, TRPV4, and TRPC6 channels) are involved in lung injury [27]. In pulmonary endothelial cells, Ca^2+^ influx through TRPC6 increases cellular permeability [28]. However, TRPM4 channels are Ca^2+^-activated nonselective cation channels (CANs) that are permeable only to monovalent ions (K^+^ and Na^+^) [29]. Therefore, it is critical to explore the relationship between the PN-OP, Ca^2+^ influx through TRPC6, and monovalent ion flux from TRPM4 channels in ARDS.

In the present study, we constructed vimentin tension probes based on fluorescence resonance energy transfer (FRET) to detect transmembrane OP gradients in live cells. We also explored the mechanism underlying intracellular or extracellular PN-OP in pulmonary edema. The results suggested that the PN-OP gradient induced by intracellular inflammasomes or extracellular blood albumin could be a new therapeutic target in ARDS and COVID-19.

## Materials and methods

### Cell culture

The A549 cell line was obtained from the American Type Culture Collection (ATCC, Manassas, VA, USA). The HULEC-5a cell line was gained from Finetest Biomart (Wuhan, China). A549 and HULEC-5a cells were cultured in Dulbecco’s Modified Eagle’s Medium (Gibco, Grand Island, NY, USA) containing 10% fetal bovine serum (FBS; Gibco), 100 μg/mL penicillin, and 100 μg/mL streptomycin (Gibco), respectively. Cells were maintained in 5% CO_2_ in a humidified atmosphere at 37 °C.

### Antibodies, reagents, and small interfering RNA (siRNA) design

Rabbit anti-NLRP3 antibodies were obtained from Affbiotech (Liyang, China). Mouse anti-apoptosis-associated speck-like (ASC) antibodies were purchased from Santa Cruz Biotechnology (Santa Cruz, CA, USA). Rabbit anti-Caspase-1 and mouse anti-glyceraldehyde-3-phosphate dehydrogenase (GAPDH) antibodies were purchased from Proteintech (Wuhan, China). Rabbit anti-α-E sodium channel epithelial 1 subunit alpha (ENaC) antibodies were obtained from Bioss (Beijing, China). Mouse anti-α-tubulin antibodies were from Boster (Wuhan, China). Horseradish peroxidase (HRP)-labeled anti-rabbit and anti-mouse antibodies were purchased from Zsgb-Bio (Beijing, China). Fluorescein isothiocyanate (FITC)-Phalloidin was purchased from Solarbio (Beijing, China).

AngII, heparin, sc-75741, and Dantrolene were purchased from Shanghai Yuanye Biochemicals (Shanghai, China). PD123319, SAR73122, GSK2193874, and 2019-nCoV Spike receptor biding domain (RBD) protein were purchased from MCE (Monmouth Junction, NJ, USA). Bradykinin was obtained from Genscript (Nanjing, China). Icatibant was purchased from Shanghai Macklin Biochemical (Shanghai, China). Losartan was purchased from Ark (Beijing, China). Cytochalasin D, nocodazole, Nimodipine, jasplakinolide (JK), taxol (Tax), and sennoside A were obtained from Sigma-Aldrich (Saint Louis, MO, USA). Tranilast was obtained from Aladdin (Shanghai, China). U73122 was purchased from Selleck (Houston, TX, USA). The siRNA targeting *ASC* was constructed by GenePhrama (Shanghai, China).

### Circularly permuted stretch sensitive FRET (cpstFRET) analysis

The plasmid encoding the FRET sensor was transfected into A549 cells according to a previously reported method [13–15, 30, 31]. The effectiveness of FRET in stable monoclonal cell lines depends on the dipole angle between donor-constitutively fluorescent enhanced cyan fluorescent protein (eCFP) and acceptor-constitutively fluorescent enhanced yellow fluorescent protein (eYFP). The images at the donor emission and acceptor emission wavelengths were recorded through a Dual View 2 splitter (MAG Biosystems, Photometrics, Tucson, AZ, USA) equipped with an × 63 oil-immersion objective lens. Argon lasers at 458 nm and 514 nm were used to test the effectiveness of the donor and acceptor. The CFP/FRET ratio was calculated using the Equation 1/E = donor/acceptor.

### Measurement of cytoplasmic OP and count rate of protein particles

Cell culture medium, the osmotic solution HEPES, and the trypsin solution were all calibrated to 300 ± 10 Osm/kg. A549 cells were cultivated in 90 mm dishes. When the cell density reached > 95%, the cells were stimulated with drugs for a certain duration. The cells were then digested and suspended in HEPES isosmotic solution and transferred to 1.5 mL Microcentrifuge tubes. Centrifugation (13,000×*g*, 5 min, 4 °C), ultrasonification (75% amplitude, 5 times, 5 s; Sonics and Materials, Connecticut, CT, USA) and another centrifugation (13,000×*g*, 10 min, 4 °C) were performed. Then, 50 µL of the supernatant solution (cytoplasm) was placed into 0.5 mL test tubes. Before use, the Osmomat 3000 Freezing Point Osmometer and 050 Membrane Osmometer (Gonotec, Berlin, Germany) were calibrated three times. The cytoplasmic OP was then measured. The kilocycles per second (Kcps) of cytoplasmic nanoparticles were detected using a Nanosight NS300 (Malvern Instruments, Malvern, UK).

### Western blotting

Cells were lysed using Radioimmunoprecipitation assay (RIPA) lysis buffer. Total proteins were extracted, and the concentrations of the proteins were analyzed using a bicinchoninic acid (BCA) detection kit (Beyotime, Shanghai, China). Protein samples were separated by 10% SDS-PAGE and transferred to polyvinylidene fluoride (PVDF) membranes.

Membranes were blocked using 5% non-fat milk for 2 h and then incubated with specific primary antibodies overnight at 4 °C. After incubation with specific secondary antibodies for 2 h, the immunoreactive protein bands were visualized using the enhanced chemiluminescent (ECL) chromogenic substrate and quantified by densitometry (Quantity One; Bio-Rad, Hercules, CA, USA). Actin or Tubulin was used as a negative control.

### Immunofluorescence (IF)

Cells were seeded in 90 mm dishes. When the cells grew to 70–80% confluence, the medium were removed and the cells were treated with different drugs for a certain duration. Cells were then fixed using 4% paraformaldehyde for 30 min. 2% Triton-100 was then applied for permeabilization, and the cells were incubated with antibodies at 4 °C overnight. Washing with phosphate-buffered saline (PBS) was followed by the addition of goat anti-mouse-FITC secondary antibody (1:200 dilution), goat anti-mouse-Tetramethylrhodamine (TRITC) secondary antibody (1:200), and FITC-phalloidin and incubated for 2 h at room temperature in the dark. Finally, 4′,6-diamidino-2-phenylindole (DAPI) was added to stain the cell nuclei. Fluorescent cells were observed under a confocal laser scanning microscope (SP5; Leica, Wetzlar, Germany).

### Measurement of intracellular calcium, chloride, and sodium ions

Enhanced NaTrium Green-2 AM (ENG; Shanxi, China), *N*-[ethoxycarbonylmethyl]-6-methoxy-quinolinium bromide (MQAE; Beyotime, China), and calcium-sensitive dye Fura-2 AM (Molecular Probes, Abcam, Cambridge, MA, USA) were used to detect intracellular sodium, chloride, and calcium ion concentrations, respectively. A549 cells were incubated with 5 μM MQAE for 30 min at 37 °C and washed 5 times with Krebs-HEPES buffer, while cells were incubated with 5 μM Fura-2 AM for 30 min at 37 °C, and then treated with Hanks’ balanced salt solution (HBSS) and incubated for another 30 min. A549 cells were incubated with 4 μM ENG-AM for 30 min at 37 °C, then treated with DMEM containing 1% FBS and incubated for another 30 min. The fluorescence intensity was detected using fluorescent microscopy (Thunder; Leica), and fluorescence images were obtained every 60 s. The normalized value of ENG fluorescence intensity (Ft/F0), MQAE fluorescence intensity (F0/Ft), and Fura-2 AM fluorescence intensity (Ft/F0) was calculated based on the ion fluorescence just after (Ft) or before (F0) the application of stimulation for 15 min, respectively.

### Caspase-1 activity assay

The Caspase-1 Inflammasome Assay (Beyotime) measured activated caspase-1 in the cultured cells and lung tissues. The cells were seeded into a 6-well plate and treated with drugs for a certain duration. The cells were then digested and suspended in lysis buffer, and placed on ice for 15 min. Meanwhile, lungs were lysed on ice for 15 min. Centrifugation (16,000–20,000 × *g*, 10–15 min, 4 °C), the supernatant solution was placed into 0.5 mL test tubes, and Caspase-1 activity was measured according to the manufacturer’s instructions.

### Electrophysiology

There recordings of TRPC6, TRPM4, and ENaC membrane currents in A549 cells were realized using a Multiclamp 700B amplifier with a Digidata 1550B Digitizer controlled by the PClamp 10 software (Molecular Devices, Sunnyvale, CA, USA). Recordings were obtained at room temperature in the whole-cell configuration. Cells were plated onto 9 mm poly-l-lysine-coated glass coverslips at suitable density, mounted in a chamber (Warner Instruments, Hamden, CT, USA), placed onto the stage of an inverted microscope, and perfused with the required solution. The details of the patch-clamp experiments are described elsewhere [32].

The standard extracellular solution contained (in mM): 145 NaCl, 5 KCl, 1 CaCl_2_, 1 MgCl_2_, 10 glucose, and 10 HEPES, adjusted pH to 7.4 with NaOH. (310–320 mOsm). The membrane potential was measured by switching the amplifier to the current-clamp mode. The standard intracellular solution contained (in mM): 140 KCl, 5 NaCl, 1 CaCl_2_, 1 MgCl_2_, 10 glucose, and 10 HEPES (pH 7.3, 290–300 mOsm). The intracellular solution for TRPC6 recordings contained (in mM): 140 CsCl, 2 MgCl_2_, 1 CaCl_2_, 10 HEPES, 10 EGTA, and 2 Na_2_ATP (pH 7.3, 290–300 mOsm). The intracellular solution for TRPM4 recordings contained (in mM): 140 CsCl, 2 MgCl_2_, 1 CaCl_2_, 10 HEPES, 10 EGTA, and 4 Mg-ATP (pH 7.3, 290–300 mOsm). The intracellular solution for ENaC recordings contained (in mM): 140 CsCl, 2 MgCl_2_, 1 CaCl_2_, 10 HEPES, 10 EGTA, and 4 Mg-ATP (pH 7.3, 290–300 mOsm).

### Measurement of transendothelial electrical resistance in the lung endothelium and epithelial cells

Transendothelial electrical resistance (TEER) was measured using an epithelial voltohmmeter (EVOM, World Precision Instruments, Sarasota, FL, USA). A549 and HULEC cells were seeded in Transwell apparatuses with a polycarbonate membrane, which were placed in a 6-well cell culture cluster, and cultured with 600 μL DMEM in the upper chamber, with 1 mL of DMEMs added into the bottom chamber. After 24 h of incubation, two Millicell^®^ ERS-2 Voltohmmeter electrodes (Merck, Kenilworth, NJ, USA) were placed on the upper and bottom chambers vertically and immersed in the medium. The TEER value was measured as a blank electrical resistance value (TEER_blank_). AngII, BK, recombinant Spike Protein RBD, and drugs were added to the upper chamber, and cells were incubated for 90 min and 24 h; the control group received the same volume of PBS. The TEER values in each chamber (TEERc) were measured. The TEER values of monolayer cells were calculated using the equation (TEERc− TEER_blank_) × S (selective membrane area) = TEER (Ω cm^2^). At least three replicates were measured for each experiment. The results are expressed as means ± SEM.

### Measurement of Na, K-ATPase activity

Cells and mouse lung tissues were digested, subjected to centrifugal sedimentation, lysed, and homogenized. Na, K-ATPase activity was measured using a minimal ATP enzyme test kit (Nanjing Jiancheng Bioengineering Institute, Nanjing, China) following manufacturer’s instructions.

### Animals

Pathogen-free, 6-to-8-week-old male C57BL/6 mice were obtained from Jiangsu Huasino Pharmaceutical Technology Co., Ltd (Jiangsu, China). The mice were raised using standard protocols: Temperature 22–25 °C, relative humidity 50–60%, and a 12 h light–dark cycle. All animals were given food and water in a standard laboratory diet. All animals were treated in accordance with the institutional animal care guidelines issued by the Experimental Animal Ethical Committee of Nanjing University of Chinese Medicine, China. The study protocol was approved by the Research Animal Care Committee of Nanjing University of Chinese Medicine (202201A037).

### Angiotensin II and bradykinin-induced lung injury

Before the test protocol, angiotensin II and bradykinin were dissolved in normal saline at 100 μg/kg, separately. Acute lung injury (ALI) was induced by angiotensin II (30 μL) and bradykinin (30 μL) intratracheal instillation. Animals in the sham group received equivalent normal saline only. Briefly, animals were randomly divided into the following five groups (n = 10 per group), control mice treated with normal saline (sham group), mice treated with AngII (model group), mice treated with AngII and drugs (treatment group), mice treated with bradykinin and mice treated with bradykinin and drugs. Drugs were injected via the intraperitoneal route.

### Lung wet to dry (W/D) weight ratio

Lungs were harvested from the different groups of mice, weighed (wet weight) dried for 3 days in a 70 °C incubator, and then the dry was weight determined. The water content was calculated as a percentage using the formula:Water content = (wet weight–dry weight)/wet weight × 100%.

### Histological analysis

Mouse lungs were fixed in 4% paraformaldehyde. After dehydration in a graded series of alcohol solutions by xylene, lungs were embedded in paraffin, sectioned at 5 mm thickness, hematoxylin–eosin stained, and analyzed histologically under a light microscope.

### Immunohistochemistry

Immunohistochemistry was performed as described previously [33]. The lung sections were deparaffinized, and 3% H_2_O_2_ was used to block endogenous peroxidases. The sections were incubated in 10% normal goat serum for 60 min at room temperature. Goat anti-rabbit Caspase-1 polyclonal antibody (1:100, Proteintech) was then added to the section, and incubation was performed overnight at 4 °C. Subsequently, HRP-conjugated secondary antibodies were applied at room temperature for 1 h. Following incubation with 3,3′-Diaminobenzidine (DAB) for 10 min, while the nuclei were stained with hematoxylin. Images were obtained under a microscope (DMi8; Leica).

### Lung permeability as measured by Evans Blue extravasation

Evans blue dye (50 mg/kg) in 200 μL of 0.9% saline was injected into the mouse tail vein and allowed to circulate for 30 min. To remove intravascular Evans blue dye, we performed transcardiac perfusion with 0.9% saline solution and obtained the lungs. To extract Evans Blue from lung tissue, tissue was weighed, homogenized, sonicated in 50% trichloroacetic acid, and then centrifuged at 13,000×*g* for 10 min. The supernatant was diluted 1:4 in 100% ethanol. Then, the obtained sample was quantified spectrophometrically (absorbance 620 nm), and expressed as micrograms per milligram lung weight.

Bronchoalveolar lung lavage fluid (BALF) was collected by flushing the lungs twice via a tracheal tube with 500 μL of PBS with protease inhibitors, followed by centrifuged at 5000×*g* for 10 min. Lavaged total protein was determined using the BCA protein assay kit.

### Non-invasive lung-function measurements (unrestrained whole-body plethysmography (UWBP))

Conscious mice were placed unrestrained into WBP boxes (EMKA-WBP, France EMKA Technologies). Pressure changes were measured for 5 min across a pneumotachograph using a flow transducer to record respiratory ventilation. The flow-derived parameter analyzer outputs included: inspiration time (Ti); expiration time (Te); peak inspiration flow (PIF); peak expiration flow (PEF); end inspiratory pause (EIP); end expiratory pause (EEP); relaxation time (RT); breathing frequency (F); enhanced pause (Penh); expiratory flow 50 (EF50), and tidal volume (TV).

### Blood biochemical assays

Blood of mice from different groups were placed in 1.5 mL EP tubes at room temperature for 1 h, then centrifuged at 3000 r/min, 4 °C for 10 min, and the supernatant serum was analyzed for hepatic and renal function.

### Statistical analyses

The CFP/FRET ratio was calculated using ImageJ (NIH, Bethesda, MD, USA). The FRET value in each subcellular region was measured for each cell, and the average value was calculated for several cells. Images were pseudo-colored using the 16-color map in ImageJ. Data are presented as mean ± SEM. Statistical analysis was performed by using GraphPad Prism 8.0. One-way analysis of variance (ANOVA) with the least significant difference test was used to determine statistical significance, and P < 0.05 was considered significant. Each experiment was repeated at least three times, > 10 cells were imaged, and each condition was analyzed.

## Results

### Cytoskeleton depolymerization is involved partly in PN-OP in response to AngII or BK stimuli

**Fig.1:**
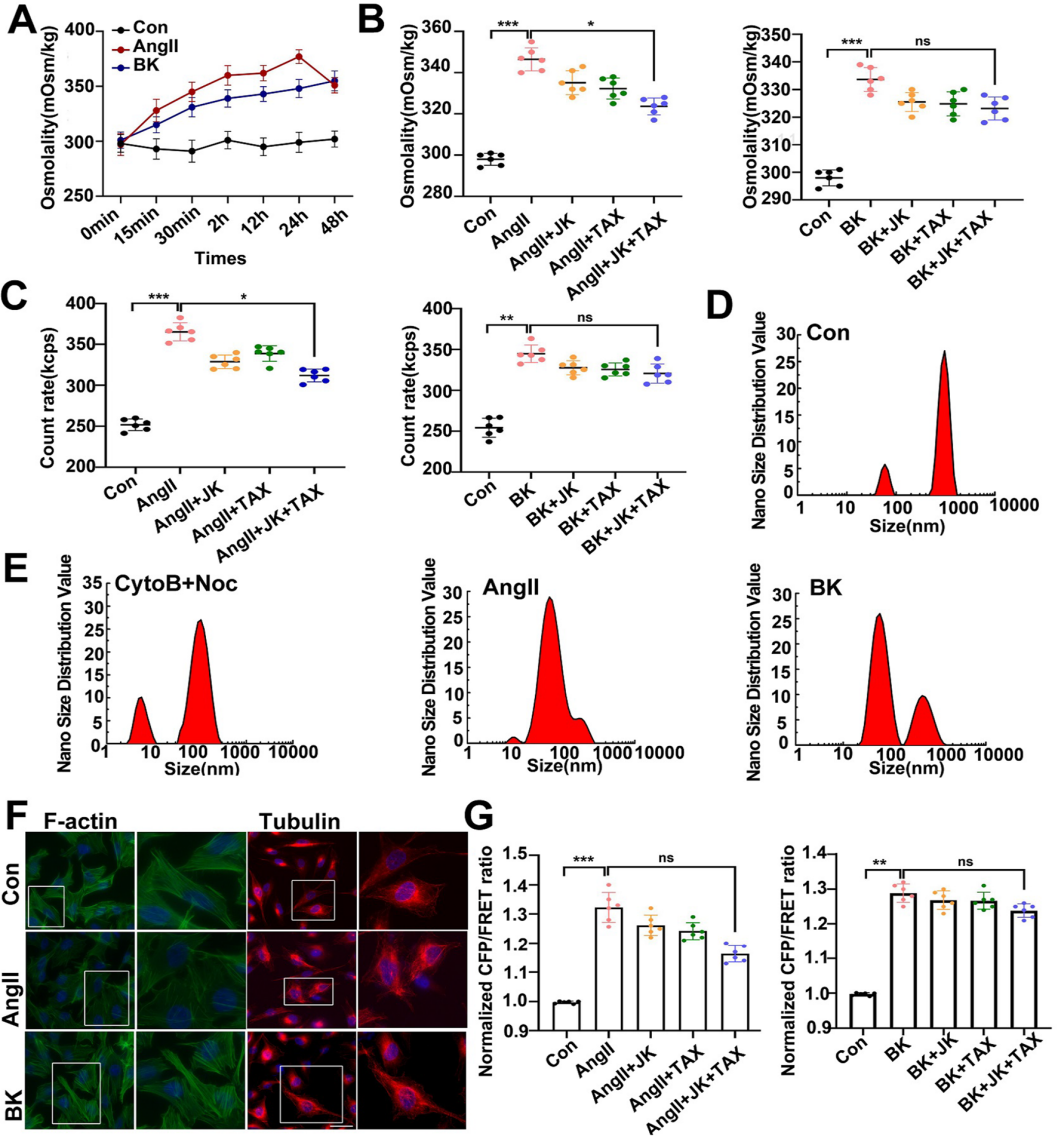
Changes of intracellular protein nanoparticle-related osmotic pressure, Vimentin tension in response to AngII or BK and treatment with cytoskeleton stabilizers. **A** Cytoplasm osmotic pressure of A549 cells were measured using a freezing point osmometer in response to AngII (10 μM) or BK (10 μM) stimuli *vs* time. **B** Cytoplasm osmotic pressure of A549 cells in response to AngII or BK, and treatment with jasplakinolide (JK, 1 μM), taxol (TAX, 10 μM), or both agents. **C** The count rate of PN in A549 cells. **D–E** Nano size distribution of protein granules in the cytoplasm. **F** Control, AngII-, and BK-treated A549 cell monolayers were stained for Phalloidin (FITC) and α-tubulin (TRITC). Images were generated from confocal laser microscopy after immunofluorescence staining. Scale bar:10 μm. **G** Normalized CFP/FRET ratios in Vimentin tension under the different treatments. Average of ≥ 5 experiments ± SEM. ns, p > 0.05, **p < 0.01, ***p < 0.001

Pulmonary edema is an important phenotype of ARDS, and ARDS is the major complication of COVID-19 [34, 35]. As reported previously, OP is the predominant factor that controls edema and is regulated by intracellular protein nanoparticles (PN) [13]. We established an alveolar epithelial swelling model using AngII or BK stimuli. The results showed a significantly increased intracellular OP under AngII (10 μM) or BK (10 μM) stimuli at different time points over 24 h (Fig. 1A). Previous studies suggested that cytoskeleton depolymerization could result in mass production of intracellular protein particles, such as β-actin and α/β-tubulin, which is involved in the occurrence of ischemia cerebral edema [13]. To explore whether or not cytoskeleton depolymerization could produce PN and induce intracellular OP increment in response to AngII and BK treatment, cells were treated with the microfilament stabilizer JK and the microtubule stabilizer Tax, alone or in combination [36, 37]. JK and Tax co-treatment partly improved the cytoplasmic OP and intracellular PN number caused by AngII. Notably, JK, TAX, or both did not alter the cytoplasmic OP and intracellular PN number under BK stimulation (Fig. 1B, C). Similarly, intracellular levels of Ca^2+^, Na^+^ and Cl^−^ could also be increased by JK and Tax co-treatment in response to AngII but not BK stimuli (Additional file 1: Fig S1A–C). In addition, we detected the size distribution of protein particles in the cytoplasm. The size distribution of most cytoplasmic particles under normal conditions was > 100 nm, and even > 1000 nm (Fig. 1D). Meanwhile, the size distribution of protein particles elicited by the cytoskeleton depolymerizer was employed as the positive control. After treatment with AngII or BK, macromolecular polymers were produced, and the distribution of cytoplasmic particles was < 100 nm and < 10 nm (Fig. 1E). The results suggest that the production of bigger PNs might be involved in regulation of intracellular OP and ions in pulmonary edema.

To verify the function exerted on the cytoskeleton under the two models, we analyzed the intracellular structural features of the cytoskeleton. AngII induced the depolymerization of cellular F-actin in the alveolar epithelium, while showing little impact on microtubules. However, BK did not alter the microfilament (MF) and microtubule (MT) structures (Fig. 1F). In addition, IF tension is closely associated with OP alteration [13, 14, 38], and JK and Tax co-treatment could partly improve IF tension caused by AngII, while JK, Tax, or both had no effect on the BK model (Fig. 1G). These results indicated a significant correlation between OP gradients and the production of abundant PN in response to AngII and BK and the increase in PN-OP partly caused by MF and MT depolymerization in response to AngII, but not BK, stimulation.

### NLRP3 inflammasomes are involved in PN-OP in response to AngII or BK stimulation

**Fig.2:**
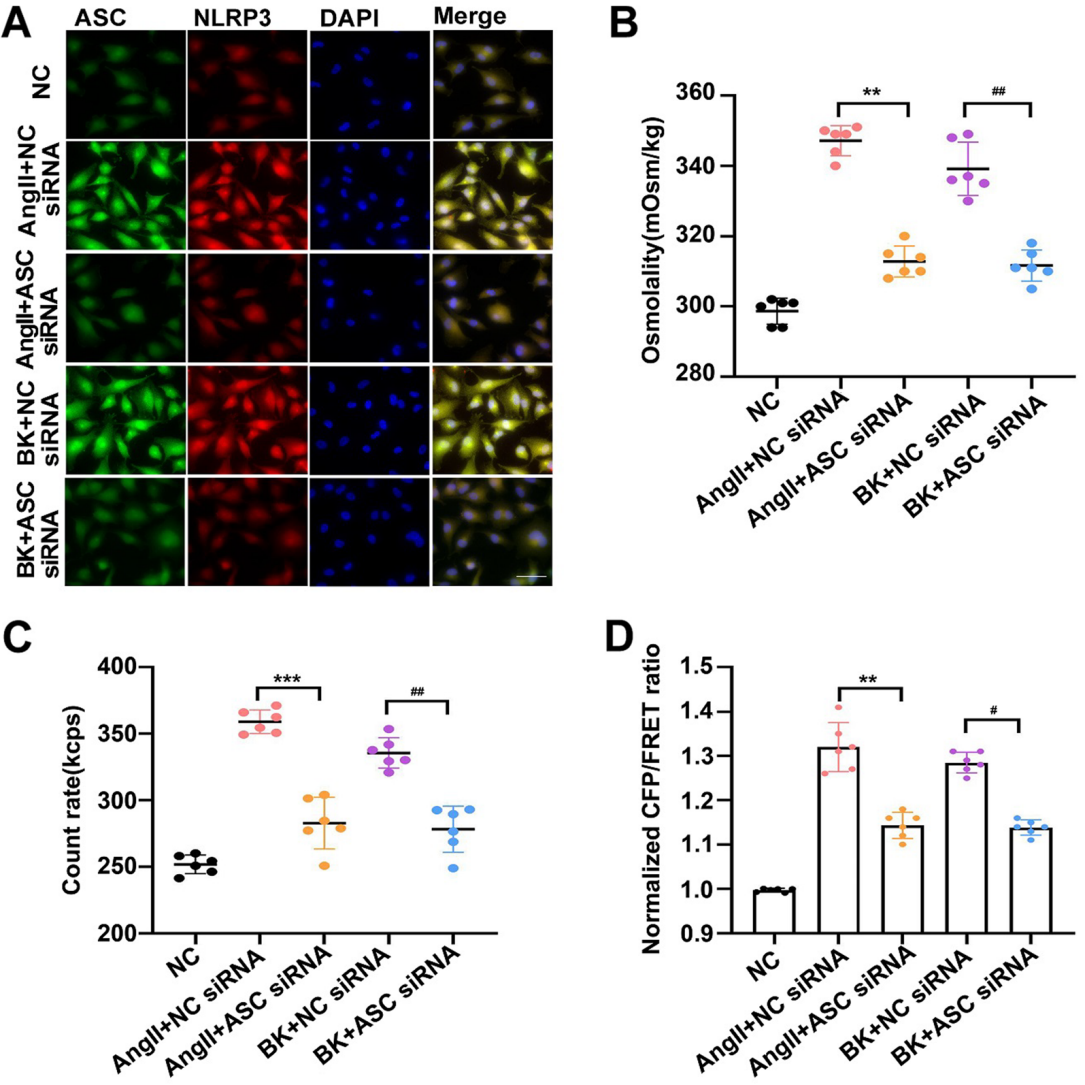
Effects of *ASC* siRNA on intracellular protein nanoparticle-related osmotic pressure and Vimentin tension in A549 cells treated with AngII or BK. **A** A549 cells were transfected with siRNA targeting the mRNA encoding ASC or empty vector. A549 cells were subjected to AngII (10 μM) or BK (10 μM) treatment alone, or co-treatment with AngII or BK and the ASC siRNA, then stained for ASC (FITC) and NLRP3 (TRITC) using antibodies. The images were generated from the fluorescence inverted microscope after immunofluorescence staining. Scale bar:20 μm. **B** Effects of *ASC* siRNA on AngII or BK-increased cytoplasmic osmolality (mOsm/kg). **C** The count rate of PN in A549 cells. **D** Normalized CFP/FRET ratio in Vimentin tension probe-transfected A549 cells at 15 min after treatment with AngII or BK treatment with the *ASC* siRNA. Average of ≥ 5 experiments ± SEM. ns, p > 0.05, **p < 0.01, ***p < 0.001

The results shown in Sect. 3.1 suggested that the production of bigger PNs is involved in PN-OP increment, and the PNs might be sourced from the production of NLRP3 inflammasomes, which are associated closely with the occurrence of ARDS [39]. NLRP3 activation is controlled by the early (10–30 min) and prolonged (> 3 h) priming pathways, respectively [40]. Thus, we explored the possible roles of both signaling pathways in PN-OP under AngII or BK stimulation. First, ASC is a crucial component of the NLRP3 inflammasome; therefore, we knocked down *ASC* via transfection of an siRNA into A549 cells to inhibit NLRP3 inflammasome assembly and activation induced by AngII and BK for 15 min. ASC oligomerization is required for NLRP3 inflammasome activation and the induction of pro-caspase-1 recruitment, as shown in Fig. 2A; therefore, when NLRP3 assembly was blocked, the accumulation of ASC specks and the levels of NLRP3 induced by AngII or BK were reduced dramatically. Meanwhile, OP (Fig. 2B), the number of PNs (Fig. 2C), and the vimentin tension (Fig. 2D), and the Ca^2+^, Na^+^ and Cl^−^ levels (Additional file 1: Fig S1 D–F) were downregulated after *ASC* siRNA transfection. We further delineated the role of nuclear factor kappa B (NF-κB) on NLRP3 inflammasome activation. Similarly, an NF-κB inhibitor also inhibited NLRP3 activation (Additional file 1: Fig S2) and decreased the OP after 24 h (Additional file 1: Fig S2). Overall, these data indicated that PNs, sourced from the production of NLRP3 inflammasomes, are involved in the upregulation of the intracellular OP under AngII and BK stimulation.

In addition, NLRP3 inhibitor MCC950 significantly inhibited MF depolymerizer (CytoB)-induced caspase-1 cleavage, production of interleukin 1 beta (IL-1β) and ASC specks (Additional file 1: Fig S3A, B and C), following with the resultant rescue of cytoplasmic OP and intracellular PN number (Additional file 1: Fig S3D, E). However, these phenomena appear not in the MT depolymerizer (Noc) group. Consistently, MF and MT stabilizers (JK and TAX) could effectively attenuated cytoplasmic OP and PN number alteration (Additional file 1: Fig S3F, G and H) in response to the LPS and nigericin. Thus, PN production induced by inflammasomes and cytoskeletal depolymerization can be regulated reciprocally in response to noxious stimuli.

### Intracellular protein nanoparticle production also resulted in hyperpolarization and VGCC-induced calcium signals

**Fig.3:**
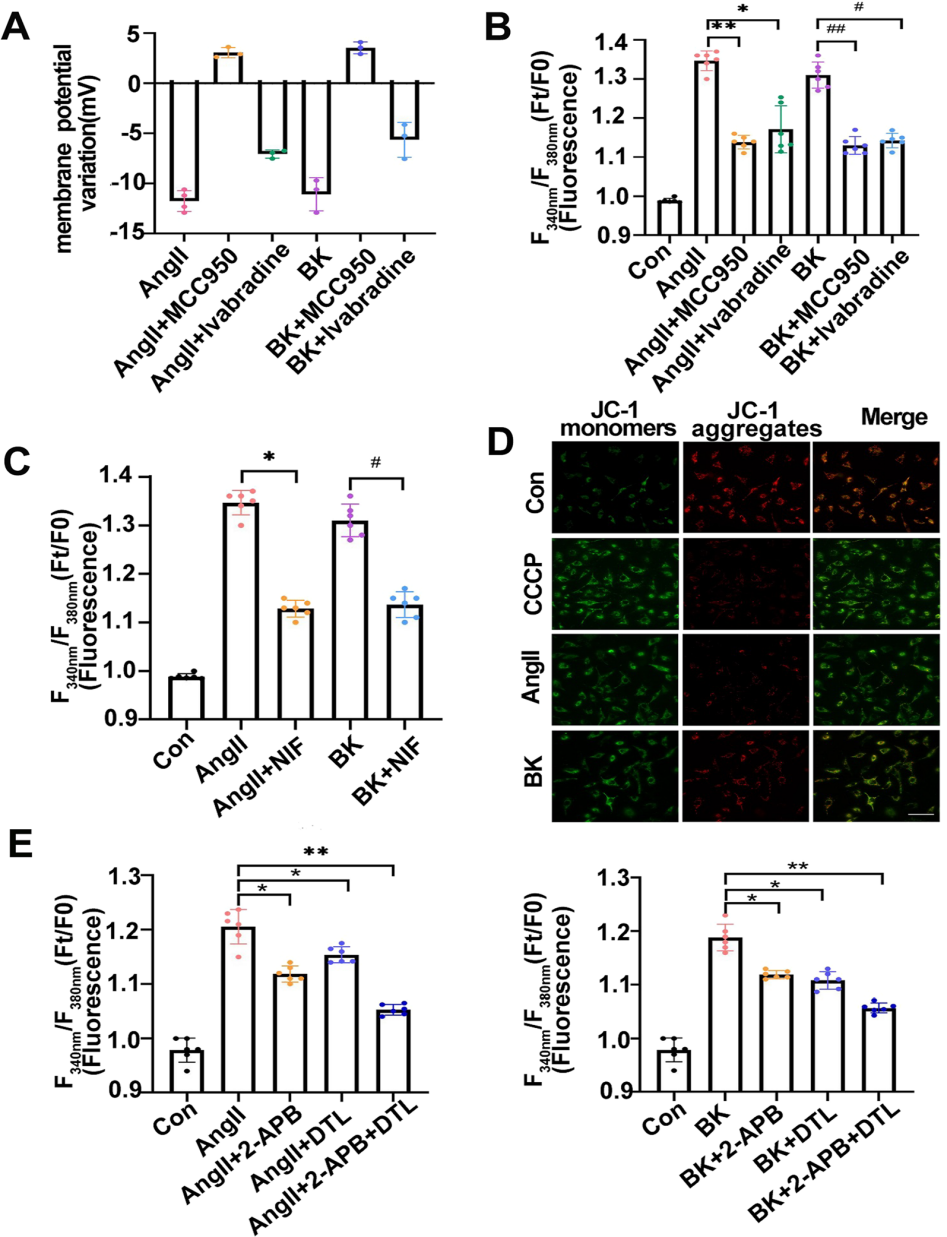
The effect of intracellular protein nanoparticles on membrane potential and cytoplasmic calcium. **A** The membrane potential was measured using the current-clamp method under AngII (10 μM) or BK (10 μM) treatment, and co-treatments of AngII or BK with MCC950 (10 μM) and Ivabradine (50 μM). **B** Changes in the fluorescence intensity (Ft/F0) of cytoplasmic calcium ions in A549 cells. **C** The membrane potential was measured using a current-clamp under AngII or BK treatment, and co-treatments of AngII or BK with Nifedipine (30 μM). **D** Photomicrograph of JC-1 fluorescence mitochondrial used to study the change in mitochondrial membrane potential in the different groups. Scale bar:20 μm. **E** Changes in fluorescence intensity (Ft/F0) of cytoplasmic calcium ions in the absence of extracellular Ca^2+^ under AngII or BK treatment, and co-treatment with 2-APB (100 μM), dantrolene (20 μM), or both agents. Average of ≥ 5 experiments ± SEM. ns, p > 0.05, **p < 0.01, ***p < 0.001

AngII or BK stimulation can result in mass PN production. According to the Donnan effect, PNs could adsorb cations and induce changes in intracellular free ion levels, which might be closely related to the membrane potential, resulting in various downstream events [41–43]. Therefore, we investigated the interaction of PN generation and voltage-gated ion channels and calcium flux in pulmonary edema. As shown in the Fig. 3A and B, both AngII and BK could cause cell membrane hyperpolarization and an increase in calcium ions, while the inflammasome inhibitor MCC950 could prevent these changes, similar to the role of the hyperpolarization-activated cyclic nucleotide-gated (HCN) channel antagonist Ivabradine (Fig. 3A and B). Meanwhile, the L-voltage-dependent calcium channel (L-VGCC) inhibitor, nifedipine, reduced Ca^2+^ influx (Fig. 3C). Next, we examined the effect of PNs on intracellular calcium ions. In a calcium-free environment, the endoplasmic reticulum (ER) is the primary source of intracellular calcium. The mitochondrial membrane potential was decreased in response to AngII or BK-induced PN increment (Fig. 3D and E). To explore the different types of Ca^2+^ channels that affect the transmembrane OP gradients under AngII and BK stimuli, we used a selection of Ca^2+^-channel inhibitors. The inhibitors included 2-APB (an inositol 1,4,5-trisphosphate receptor type 1 (IP_3_R) inhibitor), Heparin (an IP_3_R inhibitor), dantrolene (a ryanodine receptor (RyR) inhibitor), and Dizocilpine (an N-methyl-D-aspartate receptor (NMDAR) antagonist). Vimentin tension could be weakened, and the cytoplasmic OP decreased, when Ca^2+^ flux in ER was inhibited (Additional file 1: Fig S4). The above results suggested that the PN-induced alteration of membrane potential is involved in increased calcium signals via various calcium channels after AngII or BK stimulation.

### Diacylglycerol (DAG) induced calcium signaling via non-selective cation channel TRPC6 is different to the function of intracellular PN production

**Fig.4:**
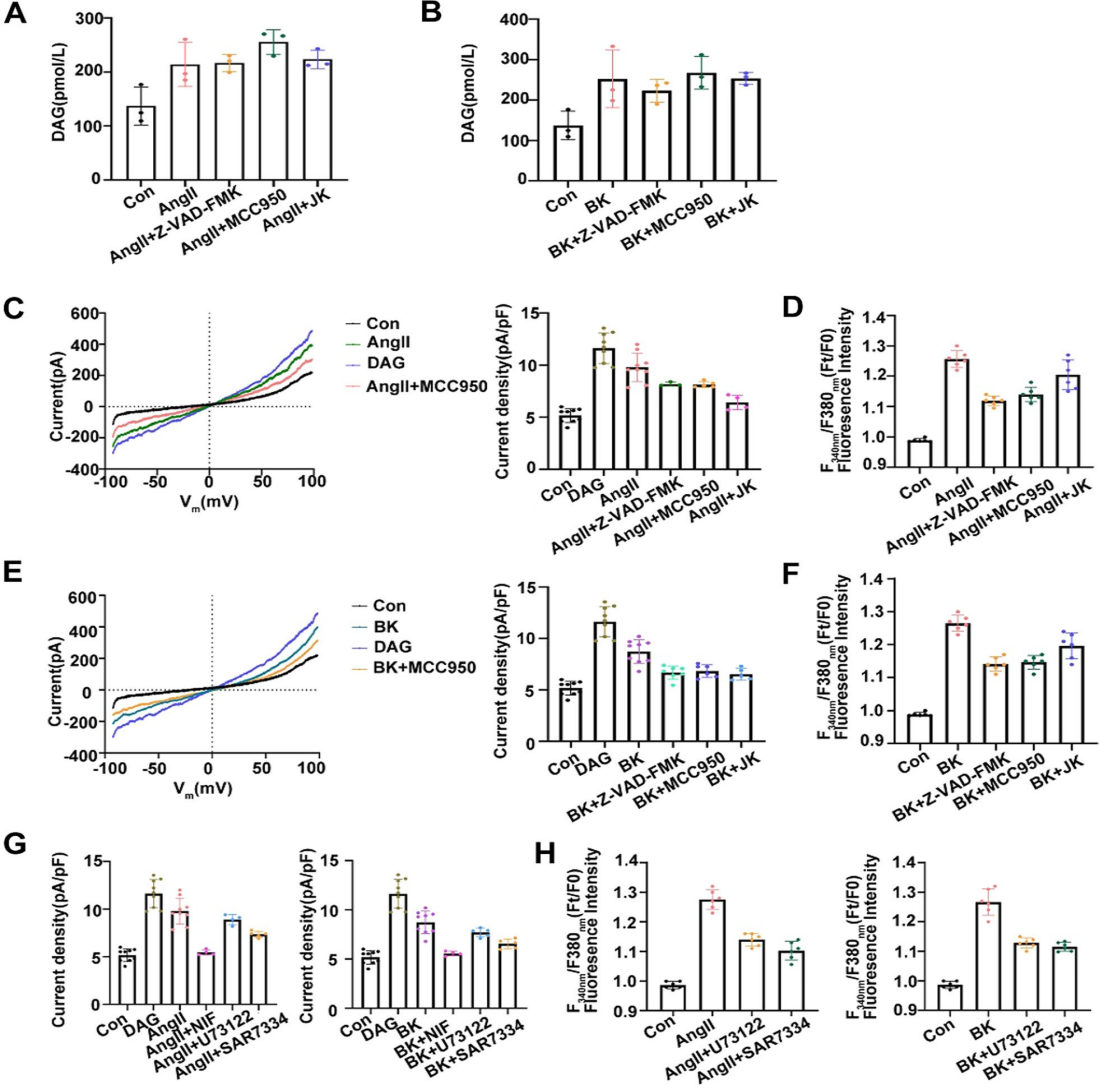
Effects on DAG-dependent activation of TRPC6 activation and calcium ions under different treatments. **A**, **B** Diacylglycerol (DAG) generation was measured under AngII (10 μM) or BK (10 μM) treatment, and AngII or BK with MCC950 (10 μM), Z-VAD-FMK (20 μM), and JK (1 μM). **C**–**G** Electrophysiological analysis of whole-cell currents in A549 cells. The voltage-ramp protocol for − 100 mV to 100 mV at a holding potential of 0 mV was given at 3 s intervals. Steady state current densities averaged at potentials of + 80 mV were extracted from the data. Representative current–voltage relationships during application of diacylglycerol (DAG, 100 μM), AngII (10 μM) or BK (10 μM), AngII or BK with MCC950 (10 μM), Z-VAD-FMK (20 μM), and JK (1 μM). Summaries of current densities–voltage relationships at holding potentials of + 80 mV. **D**–**H** The relative Fura-2 fluorescence intensity (Ft/F0) of cells under different treatments. Average of ≥ 5 experiments ± SEM. ns, p > 0.05, **p < 0.01, ***p < 0.001

Calcium signals can also be modified by ligand-gated ion channels [44]. We next examined whether or not DAG production is involved in increases in [Ca^2+^]_i_ and PN-OP through activation of the nonselective calcium channel TRPC6. An NLRP3-selective inhibitor (MCC950, 10 µM), a caspase-1 inhibitor (Z-VAD-FMK, 20 µM), or an MF stabilizer (JK, 1 µM) were used to block PN production. Decreased numbers of PNs had no effect on the DAG content after AngII or BK stimulation (Fig. 4A–B). To investigate the potential effect of AngII or BK on TRPC channels, A549 cells were used for whole cell patch recording. The currents of TRPC6 were activated by DAG. We found that reducing the numbers of PNs could decrease the TRPC6 current induced by AngII or BK and affected the time-course development of the current, reducing Ca^2+^ inflow (Fig. 4C–F, Additional file 1: Fig S5). In addition, we determined whether the L-VGCC inhibitor, Nifedipine (NIF), could affect AngII or BK-induced TRPC6 activity. As seen in Fig. 4G, NIF also blocked TRPC6 currents and Ca^2+^ influx. U73122, an inhibitor of phospholipase C (PLC), was also shown to block the activation of TRPC6 currents [45]. However, the TRPC6 inhibitor SAR7334 had more effect on Ca^2+^ level than the PLC inhibitor U73122 (Fig. 4H). The data suggested that DAG-induced non-selective activation of TRPC6 mediates calcium inflow, which differs from the effect of PNs.

### Some calcium channels are involved synergistically in cation influx and OP upregulation in a voltage-dependent manner via SUR1-TRPM4 channels

**Fig.5:**
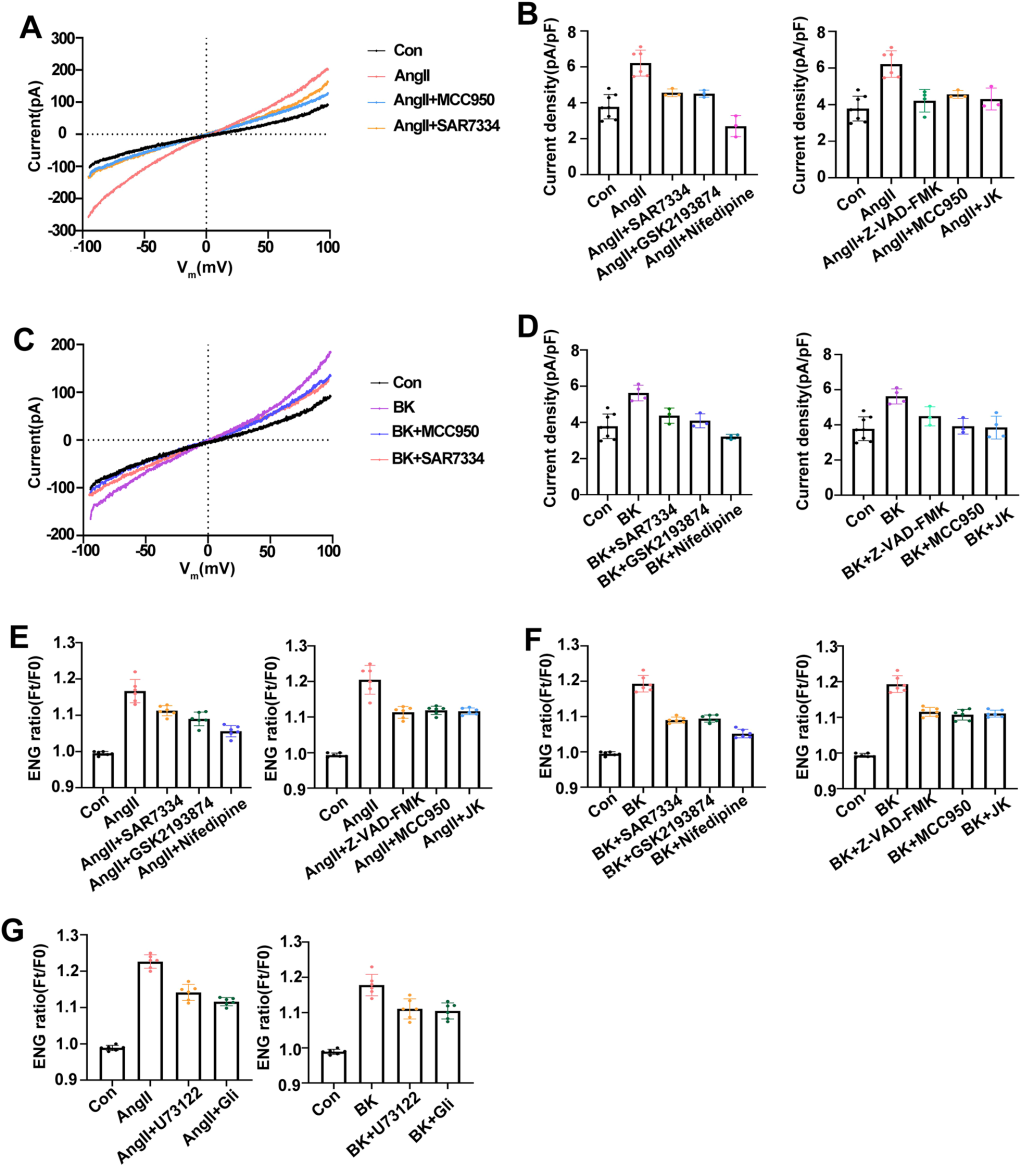
Various Ca^2+^ signals and PN production are involved synergistically in SUR1-TRPM4 activation and Na^+^ influx in response to AngII or BK stimuli. **A**, **C** Electrophysiological analysis of whole-cell currents in A549 cells. The voltage-ramp protocol for − 100 mV to 100 mV at a holding potential of 0 mV was provided at 2 s intervals. Representative current–voltage relationships during the application of AngII (10 μM) or BK (10 μM), with MCC950 (10 μM), and SAR7334 (10 μM). **B**, **D** The current densities averaged at potentials of + 80 Mv under AngII or BK, co-treatment of AngII or BK, with SAR7334 (10 μM), GSK2193874 (10 μM), Nifedipine (30 μM), Z-VAD-FMK (20 μM), JK (1 μM), and MCC950 (10 μM). **E**–**G** Changes in fluorescence intensity of ENG (Ft/F0) in cells under the different treatments. Average of ≥ 5 experiments ± SEM. ns, p > 0.05, **p < 0.01, ***p < 0.001

The nonselective ion channel TRPM4 can be activated by Ca^2+^ signals, involving mass Na^+^ influx and ion OP upregulation [46]. The TRPC6 inhibitor SAR7334, the TRPV4 inhibitor GSK193874, or L-VGCC inhibitor NIF, were used to explore the role of calcium signaling in TRPM4 activity and Na^+^ influx. Compared with the AngII or BK groups, the three Ca^2+^ inhibitors partly reduced the TRPM4 currents and intracellular Na^+^ levels, and NIF was the most effective (Fig. 5A–F). In addition, we determined whether or not PN production contributed to TRPM4 activation elicited by AngII or BK stimulation. As shown in Fig. 5A–F–Z-VAD-FMK, MCC950, and JK all reduced the TRPM4 currents and intracellular Na^+^ levels. Previous studies have shown that sulfonylurea receptor 1 (SUR1) doubles TRPM4’s affinity for Ca^2+^-calmodulin, and its sensitivity to intracellular Ca^2+^ when they co-assemble as hetromers. SUR1-TRPM4 formation can be inhibited by glibenclamide with high affinity and specificity [47]. Consistent with previous reports, glibenclamide effectively prevented SUR1-TRPM4-mediated Na^+^ influx after AngII and BK treatment (Fig. 5G). The data suggested that Ca^2+^ signals and PN production are involved synergistically in SUR1-TRPM4 activation and Na^+^ influx in response to AngII or BK stimulation.

### Drug combinations effectively reduced the PN-OP in response to AngII or BK treatment

**Fig.6:**
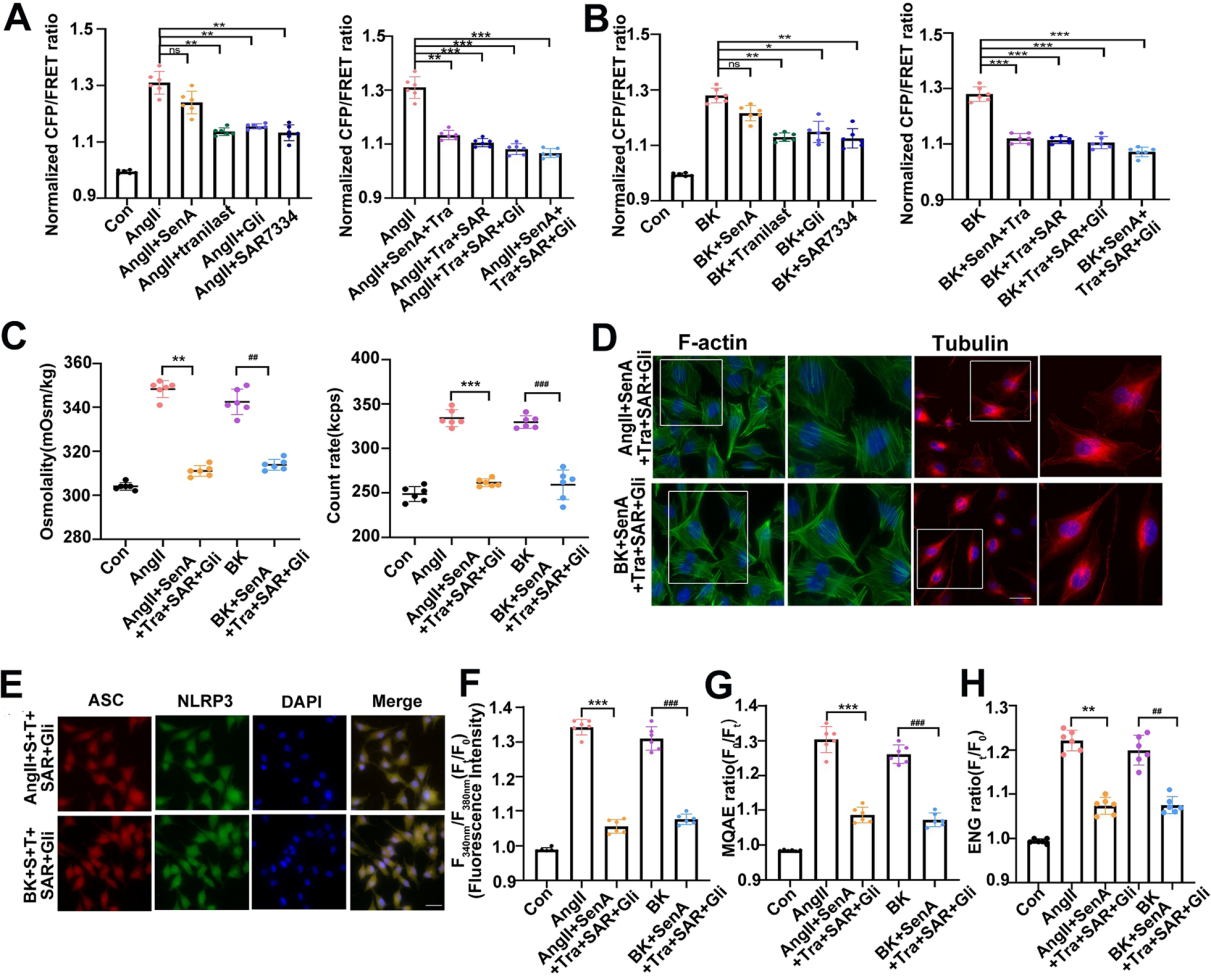
Intracellular protein nanoparticle-related osmotic pressure is reduced by effective drugs or their combinations in response to AngII or BK stimuli. **A**, **B** Mean values of normalized CFP/FRET ratios of Vimentin tension under the different treatments at 15 min. **C** Cytoplasmic osmotic pressure in A549 cells were measured after AngII (10 μM) or BK (10 μM) treatment, or with the best effective drug combinations. **D** Confocal laser microscopy observation of the immunofluorescence of Phalloidin (FITC) and α-tubulin (TRITC) in A549 cells treated with drug combinations. Scale bars, 10 μm. **E** Immunofluorescence of ASC (TRITC) and NLRP3 (FITC) in A549 cells. The images were generated from the fluorescence inverted microscope after immunofluorescence staining. Scale bar: 20 μm. **F**–**H** Changes in fluorescence intensity of cytoplasmic calcium ions (**F**), chloride ions (**G**), and sodium ions (**H**) in A549 cells subjected to AngII or BK, and AngII or BK with drug combinations. Average of ≥ 5 experiments ± SEM. ns, p > 0.05, **p < 0.01, ***p < 0.001

The intracellular OP can be upregulated by PN production, DAG-induced TRPC6 activation, and PN and Ca^2+^-induced TRPM4 opening, and is associated closely with an increment of intracellular PN, Ca^2+^, and Na^+^ levels. To explore pharmacological mechanisms underlying PN-OP in pulmonary edema, we assessed the impact of various drugs and their effective levels on the vimentin tension and PN-OP in alveolar epithelial cells after AngII or BK stimulation. Sennoside A (SenA, 100 μM), a sonic hedgehog (SSH) inhibitor, can stabilize actin filaments (Fig. 6A) [48]. Tranilast (TR; 50 μM), an anti-allergic clinical drug, is a direct NLRP3 inhibitor (Fig. 6A) [49]. Ivabradine is a blocker of HCN channels (Additional file 1: Fig S6A) [50]. NAC (50 μM), a potent scavenger of reactive oxygen species (ROS), can improve Na^+^/K^+^-ATPase activity (Additional file 1: Fig S6A) [51]. SAR7334 (10 μM), a potent and specific TRPC6 inhibitor, inhibits Ca^2+^ influx (Fig. 6A) [32]. GSK2193874 (10 μM), a TRPV4 inhibitor, can inhibit Ca^2+^ influx in a volume-dependent manner (Additional file 1: Fig S6A) [52]. NIF (30 μM), a VGCC blocker, can inhibit voltage-induced calcium influx (Additional file 1: Fig S6A) [53]. Glibenclamide (Gli, 100 μM), a SUR1-TRPM4 antagonist, contributes to decreases in intracellular sodium levels [47] (Fig. 6A). U73122 (10 μM), a PLC inhibitor, can inhibit the generation of DAG and inositol-1,4,5-trisphosphate (IP_3_) to decrease the concentration of cytoplasmic [Ca^2+^]_i_ in most cell types (Additional file 1: Fig S6A) [54]. Then, drug-combination experiments were conducted to elicit the optimal effect based on the concentrations of the single drugs. Some of these drug combinations could partly attenuate the vimentin tension and PN-OP in alveolar epithelial cells following AngII or BK stimulation (Additional file 1: Fig S6).

We showed that the most effective combination of drugs was SennosideA + Tranilast + SAR7334 + Glibenclamide (Fig. 6A and B). Meanwhile, the four drugs stated above, when given alone, also reduced the vimentin tension (Fig. 6A and B). In addition, the intracellular structure of MFs and MTs, the levels of NLRP3 inflammasomes, and intracellular Ca^2+^ and Na^+^ levels were significantly improved by the drug combination (Fig. 6C–H). However, inhibitors of HCN channels, VGCC, TRPV4, or PLC, and recovery of Na^+^/K^+^-ATPase, did not improve the effect of the four drug combination (Additional file 1: Fig S6A–D). Overall, these data suggested that inhibiting PN production by Sennoside A and Tranilast, and blocking of Ca^2+^ and Na^+^ influx by SAR7334 and Glibenclamide could effectively attenuate intracellular PN-OP in the alveolar epithelium under AngII and BK stimuli. In addition, treatment with the drug combination could effectively inhibit the increment of cytoplasmic OP, PN, IF tension (Additional file 1: FigS7A-C), and block caspase-1 cleavage, accumulation of ASC specks, and IL-1β production (Additional file 1: Fig S7D–F), along with the decreased Ca^2+^ levels and recovered cell volume (Additional file 1: Fig S7G–H) elicited by S protein. These suggested that the S protein could also induce PN-OP upregulation in alveolar epithelial cells and the drug combination might be potential therapeutics of SARS-CoV-2 infection.

### Effects of PN-OP on pulmonary permeability and alveolar fluid clearance under AngII or BK stimuli

**Fig.7:**
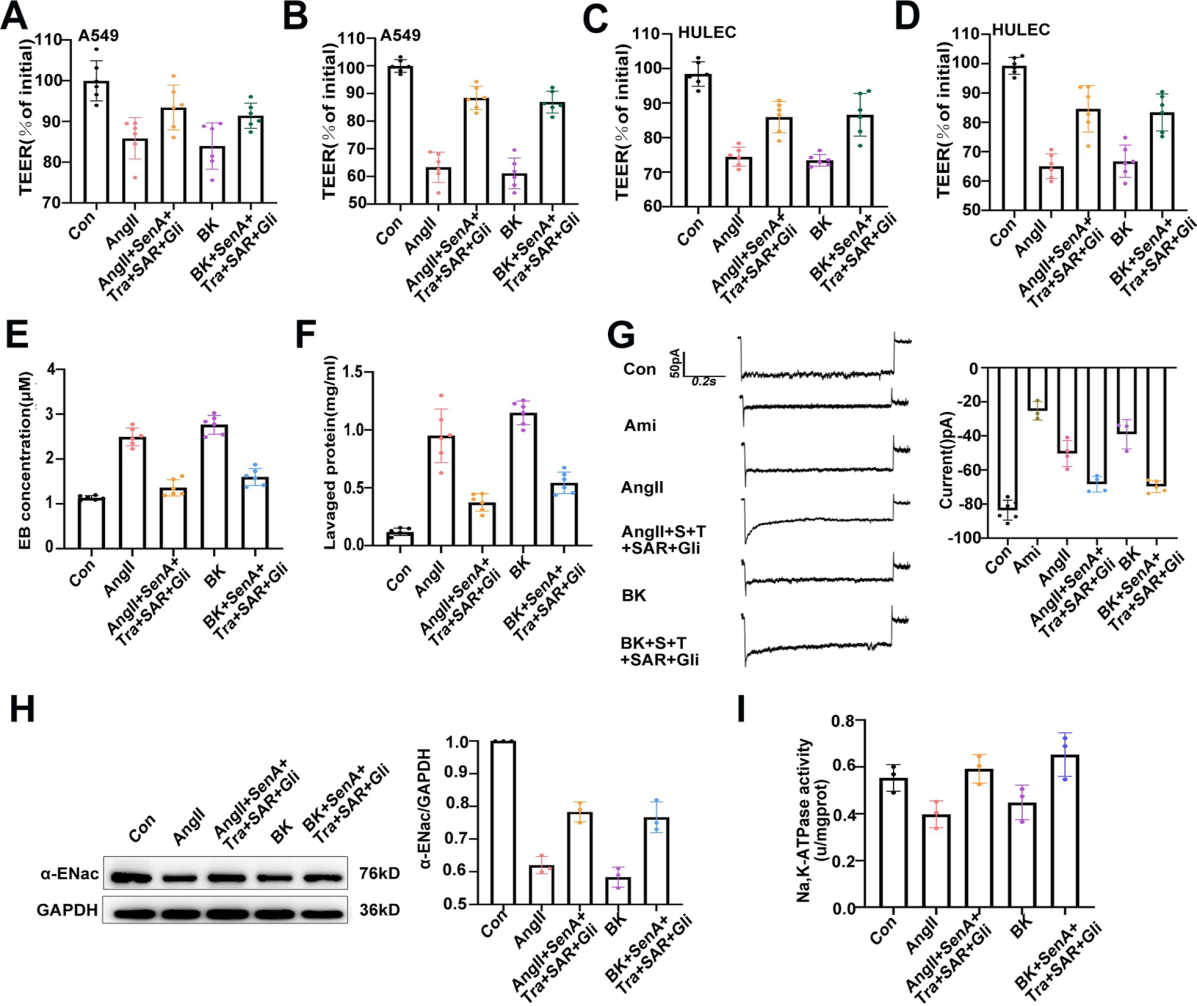
Effect of drug combinations on permeability and α-ENaC in alveolar epithelial and vascular endothelial cells stimulated with AngII or BK. **A**–**D** Normalized TEER values of each group; TEER value measured in the control group was defined as 100%. A549 and HPMEC cells were treated AngII (10 μM) or BK (10 μM), with SennosideA, Tranilast, SAR7334, and Gli for 90 min (**A**, **C**) and 24 h (**B**, **D**), respectively. **E** Quantification of EB exosmosis concentration (μM) in mouse lungs. **F** Protein concentration in bronchoalveolar lavage fluid was changed in the lungs of mice after treatment with AngII or BK and the drug combinations. **G** Summarized data for whole-cell current density elicited by a test potential at − 100 mV in the absence and presence of amiloride (10 µM). **H** ENaC-α subunit protein levels in cell lysates, as detected using western blotting. **I** Na, K-ATPase activity in A549 cells. Average of ≥ 5 experiments ± SEM. ns, p > 0.05, **p < 0.01, ***p < 0.001

In lungs affected by ARDS, pulmonary edema is thought to result from a significant increase in the pulmonary microvascular endothelial and alveolar epithelial permeability and impaired alveolar fluid clearance [5]. To examine permeability in the alveolar epithelial or endothelial swelling models following AngII or BK treatment, TEER was used to assess tight junction (TJ) formation, recorded using a conventional epithelial volt-ohm meter (EVOM). A decline in TEER across the cell monolayer of cells reflects increased permeability. We found that treatment with AngII or BK significantly reduced the TEER values over 24 h, and co-treatment with the drug combination resulted in substantially higher TEER values (Fig. 7A–D).

Next, to assess pulmonary vascular barrier function in the AngII or BK-induced mice pulmonary edema model, an Evans Blue dye assay was performed to quantify the permeability of pulmonary tissue. Under normal physiological conditions, Evans Blue, a 68 kDa protein that complexes with albumin, shows limited permeability across the vascular endothelial and alveolar epithelial barriers [55]. AngII or BK stimuli significantly increased Evans Blue accumulation in the lungs (Fig. 7E). Another method used to assess protein extravasation in another series of mice relied on the protein concentration in the BALF of the lung. As shown in Fig. 7F, compared with the sham-treated lungs, the levels of protein in the lavage substantially and significantly increased in the AngII and BK groups; however, after treatment with the drug combination, the lavaged protein level decreased dramatically. These data suggested that AngII or BK-induced PNs could increase permeability and reduce alveolar edema fluid clearance in vivo or in vitro, and recovery of the PN-OP could effectively ameliorate the changes in pulmonary microvascular endothelial and alveolar epithelial permeability.

To further understand the effect of the drug-combination on AFC under AngII and BK stimuli, we examined the protein and activity of α-ENaC as well as Na^+^, K^+^-ATPase activity [56]. In patch-clamp studies in A549 cells, AngII and BK clearly blocked ENaC ion currents, as revealed by the current density versus voltage relationship and whole-cell current (Fig. 7G). Consistent with previous reports, amiloride significantly inhibited these currents [57]. Similarly, compared with those in the control group, the levels of α-ENaC in alveolar cells were downregulated in the AngII and BK groups (Fig. 7H). Both the protein and activity of α-ENaC increased in the drug-combination groups (Fig. 7G, H). In addition, drug-combination administration significantly increased Na^+^, K^+^-ATPase activity compared with that in the AngII and BK-treated groups (Fig. 7I). These data suggested that inhibition of PN-OP promotes AFC through both of the essential mechanisms of transepithelial active sodium protein transport in the lung, mediated by ENaC and Na^+^, K^+^-ATPase.

### Effect of alveolar interstitial fluid PNs on the OP of pulmonary epithelial cells

**Fig.8:**
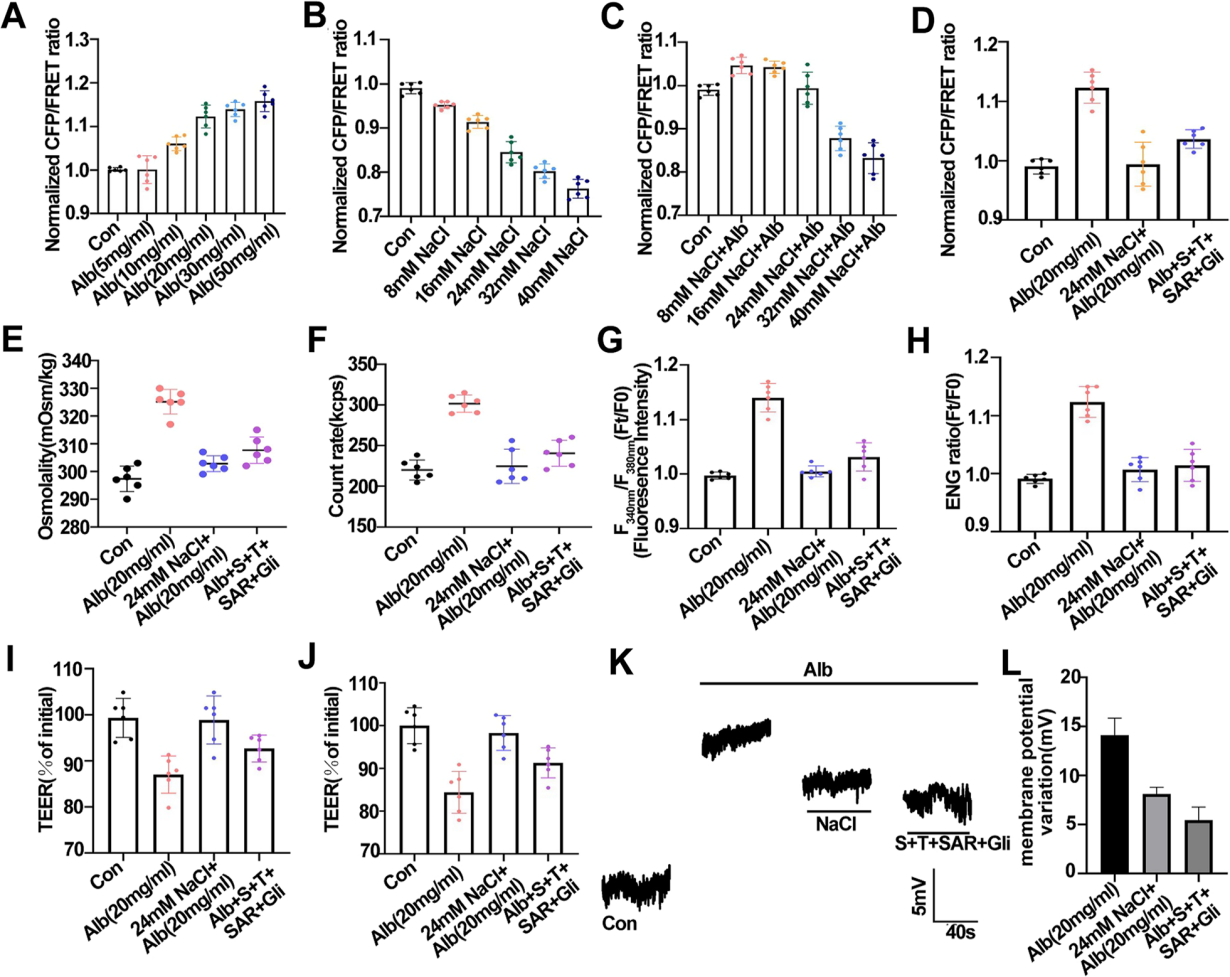
Changes in the intracellular protein nanoparticle-related osmotic pressure and vimentin tension in response to albumin, NaCl, and drug combinations. **A**, **B** Normalized CFP/FRET ratios in vimentin tension of A549 cells were measured with different concentrations of Alb (5, 10, 20, 30, 50 mg/ml) and NaCl (8,16,24,32,40 mM), respectively. **C** Normalized CFP/FRET ratios of vimentin tension under the different concentrations NaCl and Alb (20 mg/ml) co-treatments. **D** Normalized CFP/FRET ratios of vimentin tension under Alb treatment, with NaCl, drug combinations. **E** The cytoplasmic OP values. **F** The count rate of PN in A549 cells. **G**, **H** Changes in the fluorescence intensity of cytoplasmic calcium ions (**G**) and sodium ions (**H**) in A549 cells. **I**, **J** Normalized TEER values of each group in A549 cells (left) and HPMECs (right), respectively. **K**, **L** The membrane potential was measured using the Current-clamp method under Alb treatment, and co-treatments of Alb with NaCl, or drug combinations. Average of≥ 5 experiments ± SEM. ns, p > 0.05, **p < 0.01, ***p < 0.001

Among the proteins involved in protein-rich alveolar fluid, albumin, hemoglobin, clotting proteins, surfactant proteins, cytokines, and adhesion molecules disrupt the normal oncotic pressure. Some of these changes include the apparent loss of alveolar synthetic function (e.g., of dipalmitoylphosphatidylcholine (DPPC)) and an increase in plasma proteins concentrations (e.g., albumin) [58, 59]. We first examined the effect of extracellular albumin (Alb) on the vimentin tension in A549 cells. As shown in Fig. 8A, Alb treatment increased the vimentin tension in a concentration-dependent manner. In addition, according to the Donnan effect, Alb acted as an extracellular PN and adsorbed ions from the interstitial fluid of the alveoli. We found that different concentrations of NaCl decreased the IF tension (Fig. 8B), while 20 mg/ml Alb and NaCl co-treatment ameliorated the decrease in the IF tension, and 20 mg/ml Alb and 24 mM NaCl caused the IF tension to return to normal (Fig. 8C) [60]. We further assessed changes in the vimentin tension, PN-OP, and membrane potential in A549 cells under 20 mg/ml Alb treatment and co-treatment of 20 mg/ml Alb with 24 mM NaCl. Alb (20 mg/ml) treatment upregulated the cytoplasmic OP, the intracellular PN number, the calcium and sodium ion levels, membrane depolarization, and permeability (Fig. 8D–L). 24 mM NaCl antagonized the upregulation of PN-OP, intracellular Ca^2+^ and Cl^−^ levels, but only slightly affected the membrane potential (Fig. 8D–L), while Alb (20 mg/ml) and 24 mM NaCl combination treatment improved these changes (Fig. 8D–L). Based on these results, the accumulation of extracellular PNs in the alveoli fluid could result in hypertonicity of alveoli cells. The increase in extracellular ions could balance the hypertonicity of the alveoli, but also promoted the inflow of intraplasma water and exacerbated the occurrence of pulmonary interstitial fluid accumulation. Meanwhile, the results also suggested the important role of the drug combination to alter the transmembrane osmotic gradients induced by extracellular alveolar PN.

### Drug-combination treatment could improve pulmonary edema elicited by AngII or BK stimuli in vivo

**Fig.9:**
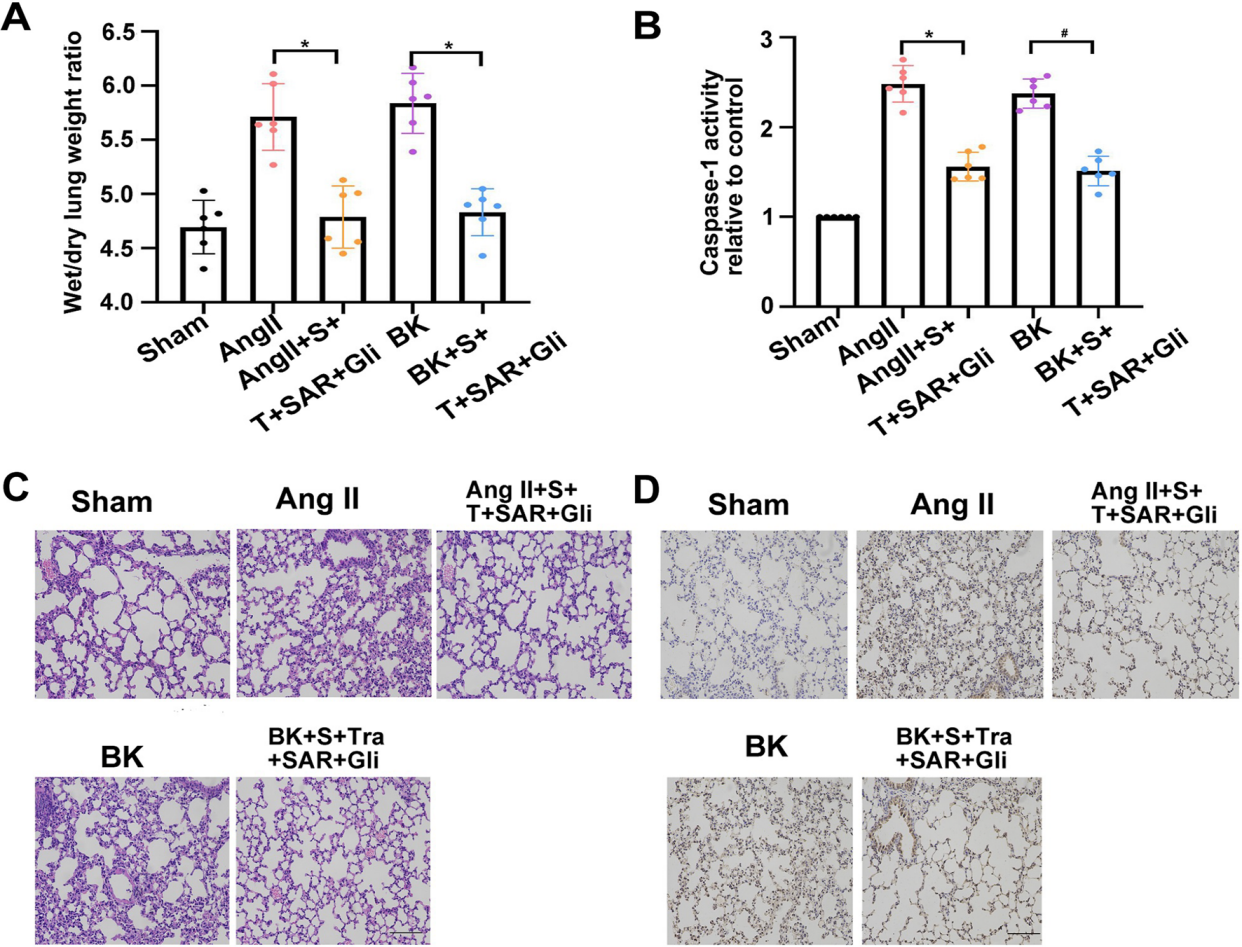
Application of drug combinations after AngII or BK-induced pulmonary edema in vivo. **A** Pulmonary wet to dry (W/D) ratio. **B** The level of caspase-1 activity significantly decreased in the serum of the drug combination group compared with that in the AngII or BK group. **C** Histology of pulmonary sections with hematoxylin and eosin staining from mice challenged with AngII or BK, which was significantly reversed after drug combination treatment. **D** IHC analyses of pulmonary tissue subjected to control, AngII (100 μg/kg) or BK (100 μg/kg), AngII or BK with drug combination stimulation. The cytoplasm was stained using caspase-1 antibodies (brown). Scale bar: 100 μm. Average of ≥ 5 experiments ± SEM. ns, p > 0.05, **p < 0.01, ***p < 0.001

To verify the mechanisms described above in vivo, we employed mouse models of pulmonary edema. First, pulmonary functional measurements were performed in mice treated with AngII or BK inhalation using uWBP. After AngII or BK inhalation, several lung capacity parameters increased significantly (Ti, Te, PEP, F, Penh, EF50, and Sr) or decreased (RT, PIF, and TV), and all these changes were effectively prevented by treatment with the drug combination (Table 1). Measurement of the water content of pulmonary tissues suggested that AngII or BK increased pulmonary edema significantly, whereas the drug combination inhibited pulmonary edema (Fig. 9A). Meanwhile, compared with that in the AngII or BK group, obvious pulmonary interstitial edema and massive infiltration of inflammatory cells were markedly attenuated in the pulmonary tissues in the drug treatment groups (Fig. 9C). Furthermore, immunohistochemical (IHC) staining and caspase-1 activity assays showed that activation of caspase-1 in pulmonary tissues and serum in the drug groups were lower than those in the AngII or BK groups (Fig. 9B and D). To detect the safety of the drug combination, we explored the changes in serum biochemistry and mouse organs. Hematoxylin and eosin (H&E) staining of the mouse organs (including the heart, liver, spleen, and kidney) revealed no significant inflammatory lesions and pathological damage in the AngII/BK groups as well as in drug combination groups (Additional file 1: Fig S8A). Moreover, we observed that AngII/BK-drug combination treatment could not change the AST, ALT, and ALP levels (liver markers) or CRE and BUN levels (kidney markers) in serum (Additional file 1: Fig S8B, C). Overall, these results suggested that the drug combination could safely and effectively mitigate AngII or BK-induced edema in vivo.

**Table 1:** Effects of drug combination treatment on altered parameters of pulmonary function under AngII or BK challenge

	Sham	AngII	AngII+S+T+SAR+Gli	BK	BK+S+T+SAR+Gli
Ti(ms)	45.32±8.19	69.57±7.21*	52.15±4.81^#^	63.17±8.89*	54.55±7.12^#^
Te(ms)	93.55±12.53	136.23±12.96**	98.18±7.79^#^	129.07±7.85*	102.26±7.81^#^
PEF(ml/s)	4.27±0.28	6.61±0.52**	5.17±0.38^#^	6.37±0.47*	5.22±0.76^#^
F(BPM)	337.89±34.82	440.81±35.01*	370.45±20.95^#^	426.06±34.29*	363.07±25.26^#^
Penh	0.41±0.24	1.08±0.17**	0.72±0.15^#^	1.23±0.39***	0.64±0.19^##^
EF_50_(ml/s)	2.86±0.35	3.78±0.36*	3.38±0.27^#^	3.55±0.17*	3.12±0.18^#^
TV(mL)	0.33±0.03	0.24±0.02*	0.29±0.03^#^	0.25±0.01*	0.28±0.04
RT(ms)	66.28±6.92	49.46±10.51^**^	59.63±7.12^#^	53.72±8.03^*^	59.57±7.46^#^
EIP(ms)	1.23±0.15	2.88±0.29**	1.62±0.11^#^	2.43±0.25^**^	1.33±0.34^##^
EEP(ms)	10.06±2.69	20.34±2.13***	13.33±1.95^###^	17.42±2.06**	13.02±1.4^###^
PIF(ml/s)	8.46±0.38	6.28±0.21*	8.13±0.52^#^	6.35±0.72*	7.67±0.47^#^
Sr(％)	13.06±3.36	24.4±5.11***	17.41±4.72^##^	26.86±5.24***	15.73±2.25^###^

*Ti* inspiration time, *Te* expiration time, *PIF* peak inspiration flow, *PEF* peak expiration flow, *EIP* end inspiratory pause, *EEP* end expiratory pause, *RT* relaxation time, *F* breathing frequency, *Penh* enhanced pause, *EF50* expiratory flow 50 and *TV* tidal volume.

Data are shown as the mean ± standard error of the mean. *p < 0.05, **p < 0.01, ***p < 0.001 for AngII or BK-challenged mice versus saline controls. ^#^p < 0.05. ^##^p < 0.01. ^###^p < 0.001 for AngII or BK/drug combination-treated mice versus AngII or BK

## Discussion

Pulmonary edema considered a typical characteristic ARDS, and is accompanied by alveolar interstitial effusion and impaired alveolar fluid clearance [5]. The OP is the predominant factor that controls edema, and it has been suggested that Ca^2+^ and Na^+^ are important triggers for intracellular OP upregulation and the development of pulmonary edema [61]. In the present study, we found that PNs are critically involved in OP regulation, being closely linked to intracellular Ca^2+^ and Na^+^ increases. Meanwhile, membrane potential alteration is an important biophysical mechanism underlying the PN-OP, involving ion channel activation and ion inflow. However, the chemical signal DAG is also involved in OP regulation through ligand-dependent TRPC6 activation and calcium signaling enhancement, which is different from the role of PN production. Thus, we propose that PN, Ca^2+^, and Na^+^ synergistically regulate the OP and its associated physical regulation of pulmonary edema, as an important pathogenetic mechanism in patients with ARDS and late-stage patients with COVID-19.

Inflammasomes, cytoskeleton depolymerization, and plasma albumin are important sources of intracellular and extracellular PNs in the lung. Activation of the inflammasome is likely to be involved in the formation of cytokine storms that drive the cascade of pulmonary inflammation, and then activate NF-κB pathways, promoting more inflammasome-based nanoparticle production [40]. Inhibition of NLRP3 activation could decrease intracellular PNs and impede the formation of the transmembrane osmotic pressure gradient, thus relieving water inflow from the plasma to the alveolar interval. A previous study indicated that depolymerization of filamentous actin (F-actin) was required for NLRP3 inflammasome activation [62]. The present study showed that disruption of the cytoskeleton exerts a pro-inflammatory effect via further activating the NLRP3/IL-1β pathway. Inflammasomes and cytoskeleton depolymerization could contribute synergistically to PN increment, OP upregulation, and pulmonary edema. Furthermore, extracellular PN accumulation also induced a change in the osmotic effect and exerted a hypertonic function via ion absorbance to maintain isotonic conditions in response to intracellular hyperosmosis [63]. Therefore, the pulmonary epithelium and alveolar epithelial cells exert a vital effect on the abnormal alteration of the OP in pulmonary edema. Inhibition of alveolar PN production and clearance of alveolar interstitial PNs (plasma proteins) was considered an effective therapeutic approach to improve the treatment of ARDS.

PNs induce hyperpolarization and depolarization of the membrane potential, both of which are involved in the activation of various voltage-dependent ion channels. The effect on the membrane potential is not only related to its potential (variation), but also is involved in the regulation of the temporal development of ion channels, such as TRPC6 and TRPM4 [45, 64]. However, PN-mediated regulation of the membrane potential is not only related to its quantity, but also is linked to the distribution of their particle size. According to the Donnan effect and the theory of double layers, only 0.1–1000 nm protein particles have an adsorption effect on cations, and their adsorption ion changes are also regulated by the high-valency ions, such as divalent calcium and magnesium ions [65], suggesting that PNs are involved in membrane potential regulation in concert with various factors. Thus, it should be a gordian knot to restore intracellular PNs and their ion adsorption to a normal state, even if cytoskeleton depolymerization and inflammasome production is inhibited, which would impede the improvement of clinical pulmonary edema.

Previous reports suggested that Na^+^ inflow is an important source of increased intracellular OP [46]. However, in the present study, we found that PN and Ca^2+^ are crucial factors for Na^+^ channel activation and Na^+^ inflow, which are related, but distinct. Specifically, PN-induced membrane potential changes can independently activate voltage-dependent Ca^2+^ and Na^+^ channels; meanwhile, the chemical signal DAG can also induce activation of ligand-dependent Ca^2+^ channels (TRPC), which is linked to potential changes, but differs from the effect of PN-induced potential changes alone, in that it more effectively activates TRPC6 channels to mediate non-selective calcium inflow. Calcium signals can activate non-selective Na^+^ channels and its inflow, which are also closely associated with membrane potential changes [64]. Thus, PNs are essential for edema control; however, they are not the only regulator of alveolar edema. Therefore, by combining multiple sources of PNs, calcium ions, and sodium ions, multi-targeted blockade is an indispensable pharmaceutical development to balance the intracellular OP and improve edema.

Intracellular ions and PNs control the transmembrane osmotic gradient and water flux [13, 14]. Based on this, inhibition of microfilament depolymerization, inflammasome production, and Ca^2+^ and Na^+^ inflow could effectively decrease the PN-OP. Intracellular PN inhibition and OP recovery could also improve the markedly abnormal membrane potential, cell volume, and ion homeostasis, which was supported by the data showing that inhibitors of HCN, L-VGCC, and TRPV4 channels and recovery agents of Na^+^/K^+^ ATPase has less effect on the improvement of OP and pulmonary edema than inhibition of PNs, TRPC6, and SUR1-TRPM4. However, PLC, as upstream signal of DAG and TRPC6, exerts less effect on the PN-OP than TRPC6, in which PIP_2_ or IP_3_ signals might be involved in improvement of intracellular ion homeostasis via other pathways [54]. Meanwhile, the drug combination also improved the PN-OP and the pulmonary dysfunction elicited by the spike protein in patients with COVID-19.

An increased PN-OP not only leads to potential changes in water metabolism, but also induces destruction of the pulmonary vascular barrier, which is associated closely with mechanical mechanisms underlying the accumulation of interstitial fluid in the lung. This viewpoint was supported by a previous report that increases in intracellular PNs and OP are involved in destruction of the blood–brain barrier function, resulting in its non-selective hyperpermeability in response to multiple chemical stimuli [15]. Therefore, alveolar PN formation is involved in the disruption of endothelial and alveolar barrier functions, which contributes to the interstitial fluid in the alveoli containing high levels of extravasated macromolecules. Extracellular high protein levels significantly increased the transmembrane OP gradient, possible increasing transendothelial and transepithelial fluid movement into the interstitial and, later, into the alveolar space [66]. In addition, the PN-OP also blocks the effusion re-absorption of the alveolar epithelium through inhibition of ENaC and Na^+^, K^+^-ATPase activities. Overall, recovery of the PN-OP could contribute to ion homeostasis and the potential balance between the pulmonary microvascular endothelium and the alveolar epithelium, suggesting that force, electrical, and chemical signals, exerted synergistically, function on live cells.

In summary, PNs dominated by inflammasomes and the depolymerized cytoskeleton play a pivotal role in pulmonary edema in ARDS and COVID-19. Intracellular PNs construct transmembrane osmotic pressure gradients among capillaries, alveolar septum, and the alveolar epithelium through hyperpolarization and calcium signaling. Meanwhile, alveolar interstitial PNs also contribute to membrane depolarization and transmembrane osmotic pressure gradients. Overall, downregulation of PN production and blockade of non-selective cation channels (TRPC6 and TRPM4) could relieve pulmonary edema in ARDS. Thus, multi-target therapy has become an essential clinical initiative to improve the treatment of pulmonary edema associated with ARDS and to treat late-stage patients with COVID-19, bringing new developments to clinical treatment.

## Supplementary Information

**Additional file 1**: **Fig S1** The relationship between cell osmolarity and intracellular ion contents. **Fig S2** Effects of NF-κB inhibitors on intracellular protein nanoparticle-related osmotic pressure and Vimentin tension in A549 cells treated with AngII or BK. **Fig S3** The interaction of MFs/MTs depolymerization and NLRP3 inflammasomes in A549 cells. **Fig S4** Effects of various Ca^2+^-channel inhibitors on intracellular protein nanoparticle-related osmotic pressure and Vimentin tension in A549 cells treated with AngII or BK. **Fig S5** Inhibition of the DAG-induced current in A549 cells under the different treatments. **Fig S6** Drug screening to reduce Vimentin tension in A549 cells treated with AngII or BK. **Fig S7** Changes in intracellular protein nanoparticle-related osmotic pressure and the potential roles of four selected drugs in response to the spike protein. **Fig S8** Safety assessment of the drug combination in vivo. (Previously, in FigS2, the D and E chart labels 'AngII', 'BK' were incorrectly written as 'Con ', and they are now corrected.)

## Data Availability

The original contributions presented in the study are included in the article/ Supplementary Material. Further inquiries can be directed to the corresponding author.

## Abbreviations

ARDS: Acute respiratory distress syndrome
PE: Pulmonary edema
OP: Osmotic pressure
IF: Intermediate filament
PN: Protein nanoparticle
COVID-19: Coronavirus 2019
ACE2: Angiotensin-converting enzyme 2
RAS: Renin–Angiotensin
KKS: Kinin–Kallikrein
AngII: Angiotensin II
BK: Bradykinin
AECs: Alveolar epithelial cells
TRP: Transient receptor potential
CANs: Ca^2+^-activated nonselective cation channels
FRET: Fluorescence resonance energy transfer
JK: Jasplakinolide
Tax: Taxol
Kcps: Kilocycles per second
TEER: Transendothelial electrical resistance
MF: Microfilament
MT: Microtubule
