# Supplementary material for: Protein nanoparticle-induced osmotic pressure gradients modify pulmonary edema through hyperpermeability in acute respiratory distress syndrome
Source: J Nanobiotechnology. 2022 Jul 6;20:314. doi: 10.1186/s12951-022-01519-1 (PMC9257569; doi:10.1186/s12951-022-01519-1)
Supplement: Supplementary file 1 — Additional file 1: Fig S1 The relationship between cell osmolarity and intracellular ion contents. Fig S2 Effects of NF-κB inhibitors on intracellular protein nanoparticle-related osmotic pressure and Vimentin tension in A549 cells treated with AngII or BK. Fig S3 The interaction of MFs/MTs depolymerization and NLRP3 inflammasomes in A549 cells. Fig S4 Effects of various Ca2+-channel inhibitors on intracellular protein nanoparticle-related osmotic pressure and Vimentin tension in A549 cells treated with AngII or BK. Fig S5 Inhibition of the DAG-induced current in A549 cells under the different treatments. Fig S6 Drug screening to reduce Vimentin tension in A549 cells treated with AngII or BK. Fig S7 Changes in intracellular protein nanoparticle-related osmotic pressure and the potential roles of four selected drugs in response to the spike protein. Fig S8 Safety assessment of the drug combination in vivo. (Previously, in FigS2, the D and E chart labels 'AngII', 'BK' were incorrectly written as 'Con ', and they are now corrected.) [file 12951_2022_1519_MOESM1_ESM.pdf]

Supplementary materials

Protein nanoparticle-induced osmotic pressure gradients modify pulmonary edema through hyperpermeability in acute respiratory distress syndrome

ZhiZhi Qian[1,2#], HuiWen Wu[3#], ZhaoShun Qiu[1,2], DanYang Li[1,2], ChenCheng Zhang[1,2], QianYi Wang[1,2], XiYu Xiong[1,2], ZiHui Zheng[1,2], QinLi Ruan[1,2], YiChen Guo[3,*], Jun Guo[1,2,*]

1 School of Medicine & Holistic Integrative Medicine, Nanjing University of Chinese Medicine, Nanjing 210023, Jiangsu, PR China

2 Key Laboratory of Drug Target and Drug for Degenerative Disease, Nanjing University of Chinese Medicine, Nanjing 210023, Jiangsu, PR China.

3 Laboratory Center for Basic Medical Sciences, Nanjing Medical University, Nanjing 211166, PR China.

[#] contributed equally to this work

* **Correspondence:** Jun Guo, School of Medicine & Holistic Integrative Medicine, Nanjing University of Chinese Medicine, Nanjing 210023, Jiangsu, China

Email: guoj@njucm.edu.cn

YiChen Guo, Biomedical Engineering, University of Alabama at Birmingham School of Medicine, Birmingham, AL, USA.

Email: yguo16@uab.edu

Supplementary Fig. 1

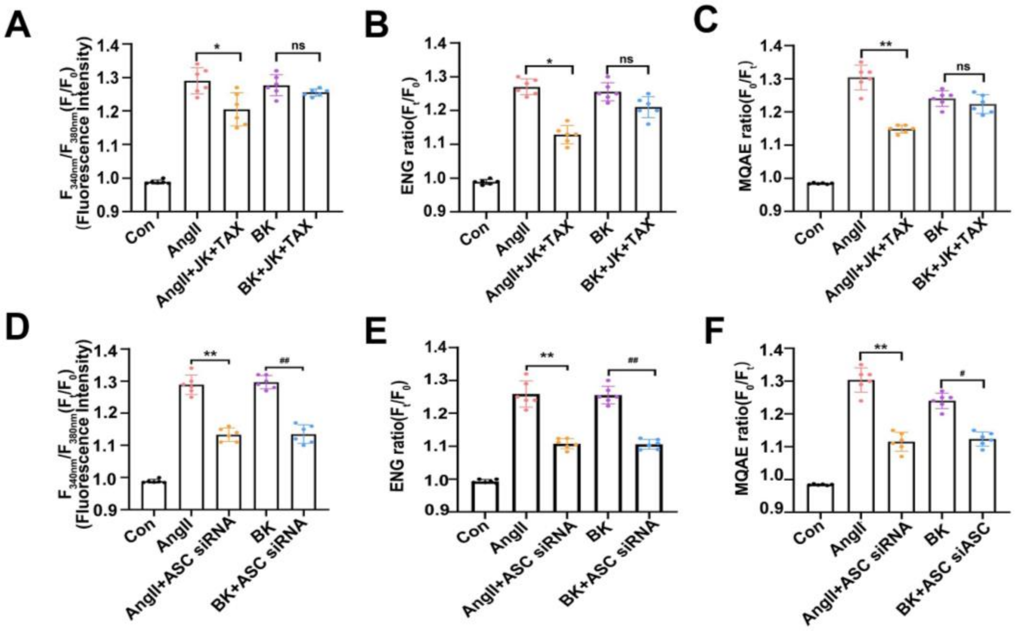

sFig.1 The relationship between cell osmolarity and intracellular ion contents. (A,D) The relative Fura-2 fluorescence intensity (Ft/F0) of cells under the different treatments. (B,E) Changes in fluorescence intensity of ENG (Ft/F0) in cells under different treatments. (C,F) Changes in fluorescence intensity of MQAE (F0/Ft) in cells. The increase of intracellular Cl^- levels led to a decrease in the MQAE fluorescence value. Average of ≥ 5 experiments ± SEM. ns, $p > 0.05$, **$p < 0.01$, ***$p < 0.001$.

Supplementary Fig. 2

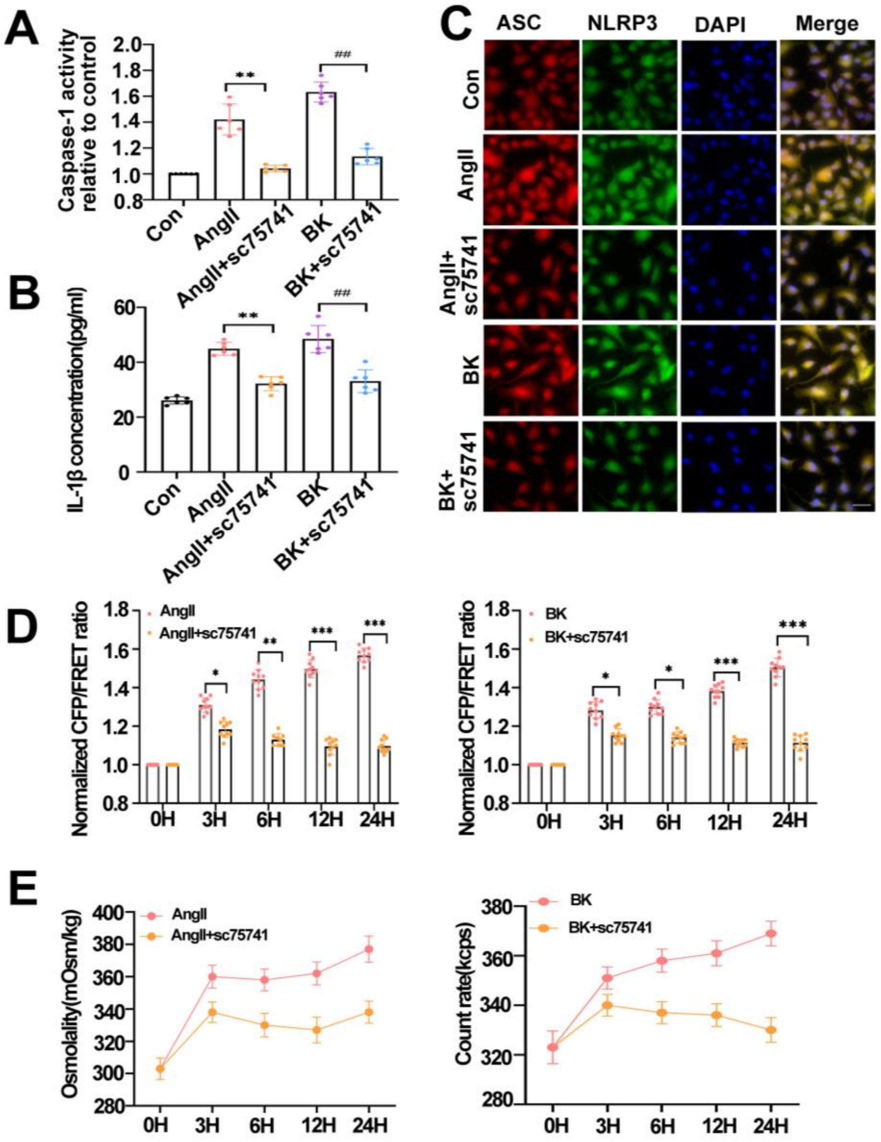

sFig.2 Effects of NF-κB inhibitors on intracellular protein nanoparticle-related osmotic pressure and Vimentin tension in A549 cells treated with AngII or BK. (A) Caspase-1 activity and (B) IL-1β release of cells in response to AngII or BK, with sc75741. (C) AngII or BK-primed-A549 cells were treated with sc75741 (20 μM). Immunofluorescence of ASC and NLRP3 in A549 cells. Scale bar: 20μm. (D) Normalized CFP/FRET ratio in Vimentin tension probe-transfected A549 cells at 15 min after AngII or BK treatment with sc75741. (E) The cytoplasmic OP value s(left) and the count rate of PN (right) in A549 cells. Average of ⩾ 5 experiments ± SEM. ns, $p > 0.05$, * *$p < 0.01$, ***$p < 0.001$.

Supplementary Fig. 3

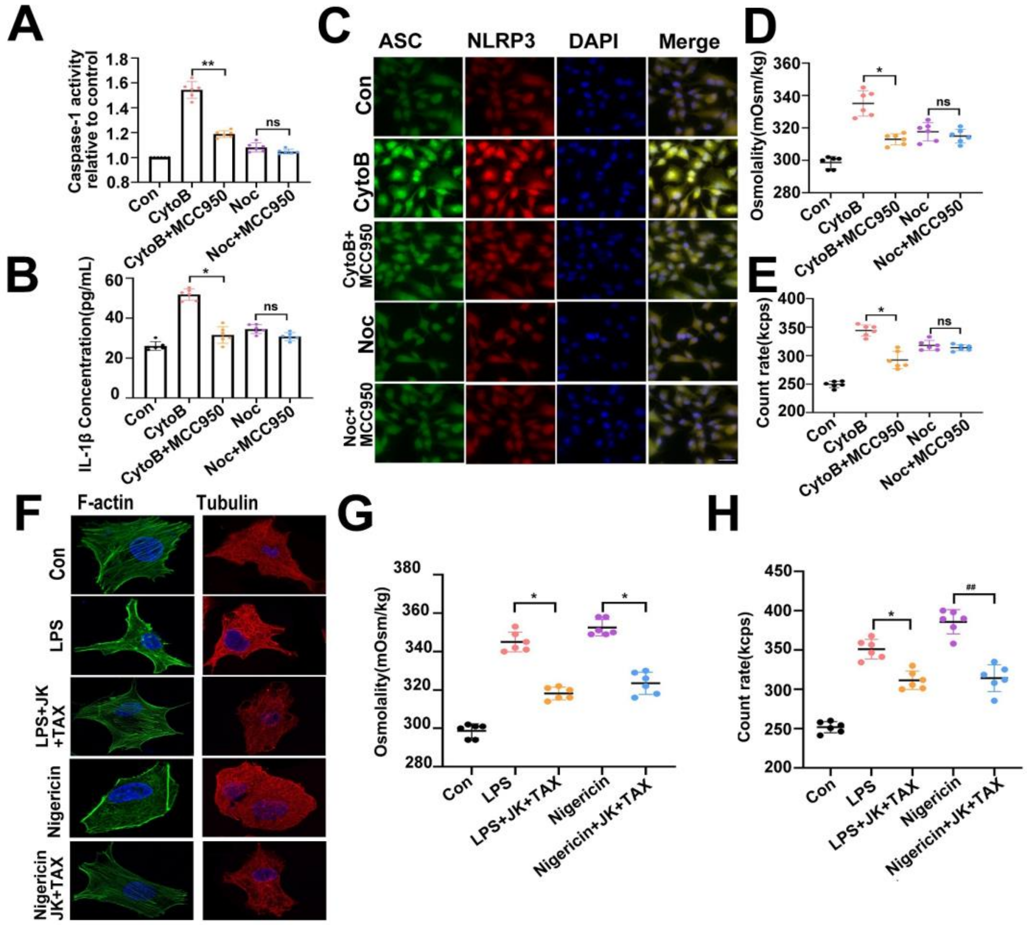

sFig.3 The interaction of MFs/MTs depolymerization and NLRP3 inflammasomes in A549 cells. (A) Caspase-1 activity and (B) IL-1β release of cells treated with CytoB, CytoB+MCC950, Noc, and Noc+MCC950. (C) Immunofluorescence of ASC and NLRP3 in A549 cells. Scale bar: 20μm. (D) The cytoplasmic OP values of A549 cells were measured using a freezing point osmometer. (E) The count rate of PN in A549 cells. (F) Monolayers of A549 cells were stained for Phalloidin (FITC), α-tubulin (TRITC) under LPS treatment, and co-treatments of LPS with JK and TAX, Nigericin treatment, and co-treatments of Nigericin with JK and TAX. (G) Cytoplasmic osmotic pressure and (H) count rate of protein nanoparticles in A549 cells were measured by LPS or Nigericin, with MF/MT stabilizers (JK and TAX). Average of ≥ 5 experiments ± SEM. ns, $p>0.05$, ** $p<0.01$, *** $p<0.001$.

Supplementary Fig.4

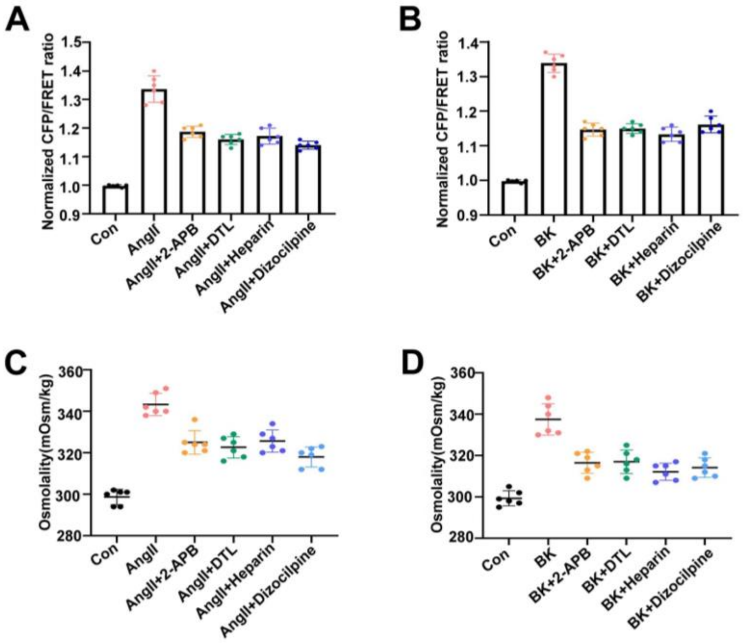

sFig.4 Effects of various Ca^{2+}-channel inhibitors on intracellular protein nanoparticle-related osmotic pressure and Vimentin tension in A549 cells treated with AngII or BK. (A-B) Normalized CFP/FRET ratios of Vimentin tension under the different treatments. (C-D) The cytoplasmic OP values of A549 cells were measured using a freezing point osmometer under the different treatments. Average of ≥ 5 experiments ± SEM. ns, $p > 0.05$, * *$p < 0.01$, ***$p < 0.001$.

Supplementary Fig. 5

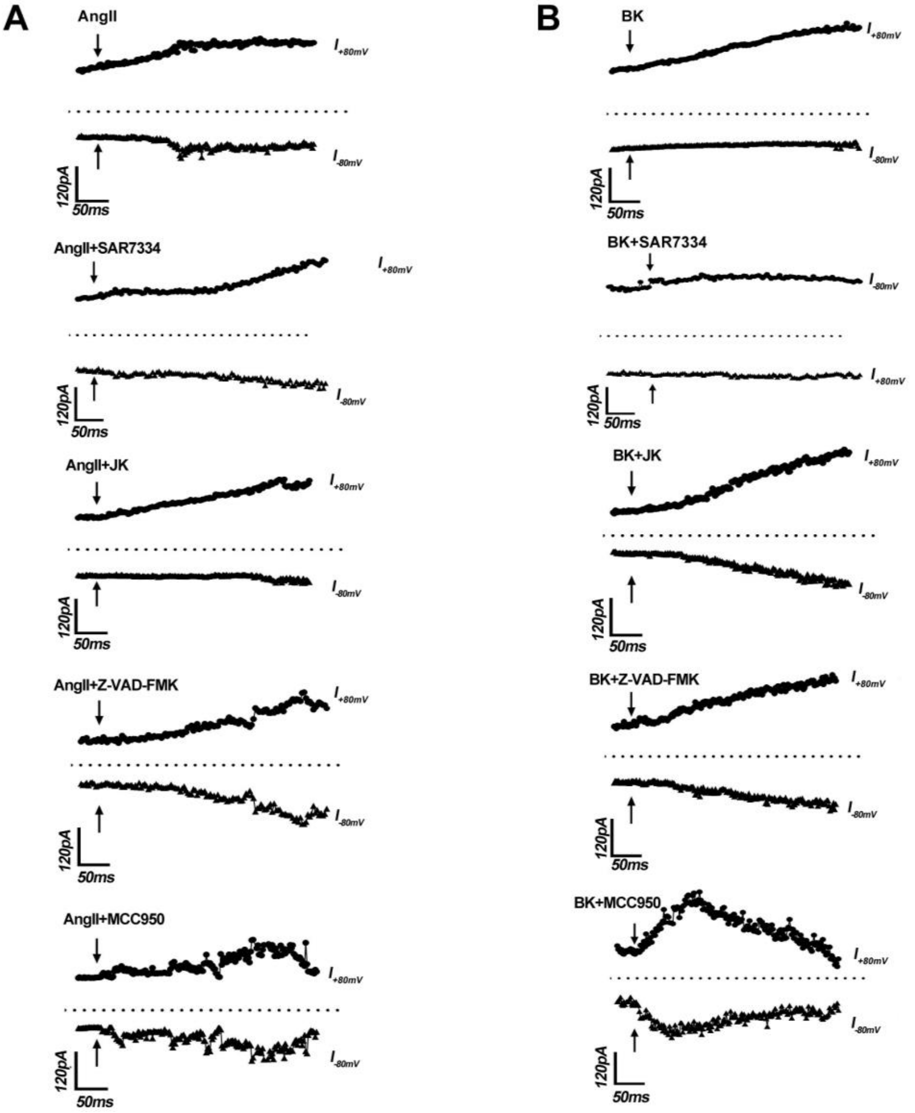

sFig.5 Inhibition of the DAG-induced current in A549 cells under the different treatments. (A-B) Electrophysiological whole-cell recordings of diacylglycerol-induced TRPC6-like currents in A549 cells. Averaged time-course development of the current in A549 cells were recorded at ± 80 mV during the application of AngII (10 μM) or BK (10 μM), AngII or BK with SAR7334 (10 μM), JK (1 μM), Z-VAD-FMK (20 μM), and MCC950 (10 μM).

Supplementary Fig. 6

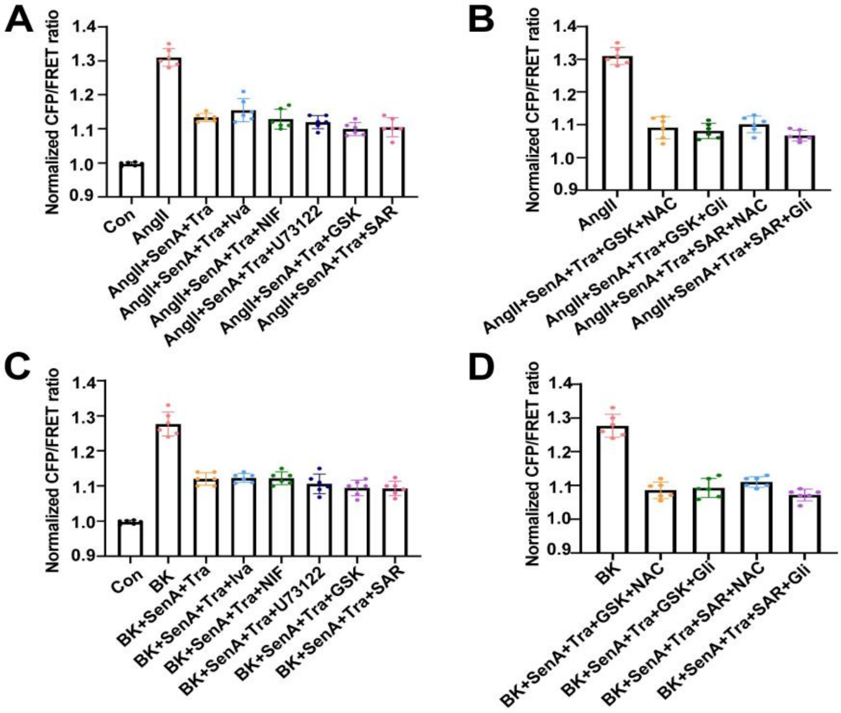

sFig.6 Drug screening to reduce Vimentin tension in A549 cells treated with AngII or BK. Normalized CFP/FRET ratios of Vimentin tension treated with the PN inhibitors SenA and Tra after subjection to (A) AngII or (C) BK, and Ivabradine, NIF, U73122, GSK2798745, and SAR7332 were also added, respectively. Normalized CFP/FRET ratios of Vimentin tension treated with the PN inhibitors SenA and Tra after subjection to (B) AngII or (D) BK alone, and SenA; Tra, GSK2798745, and NAC; with SenA; Tra, GSK2798745, and Gli; with SenA; Tra, SAR7332, and NAC, with SenA; Tra, SAR7332; and Gli.

Supplementary Fig. 7

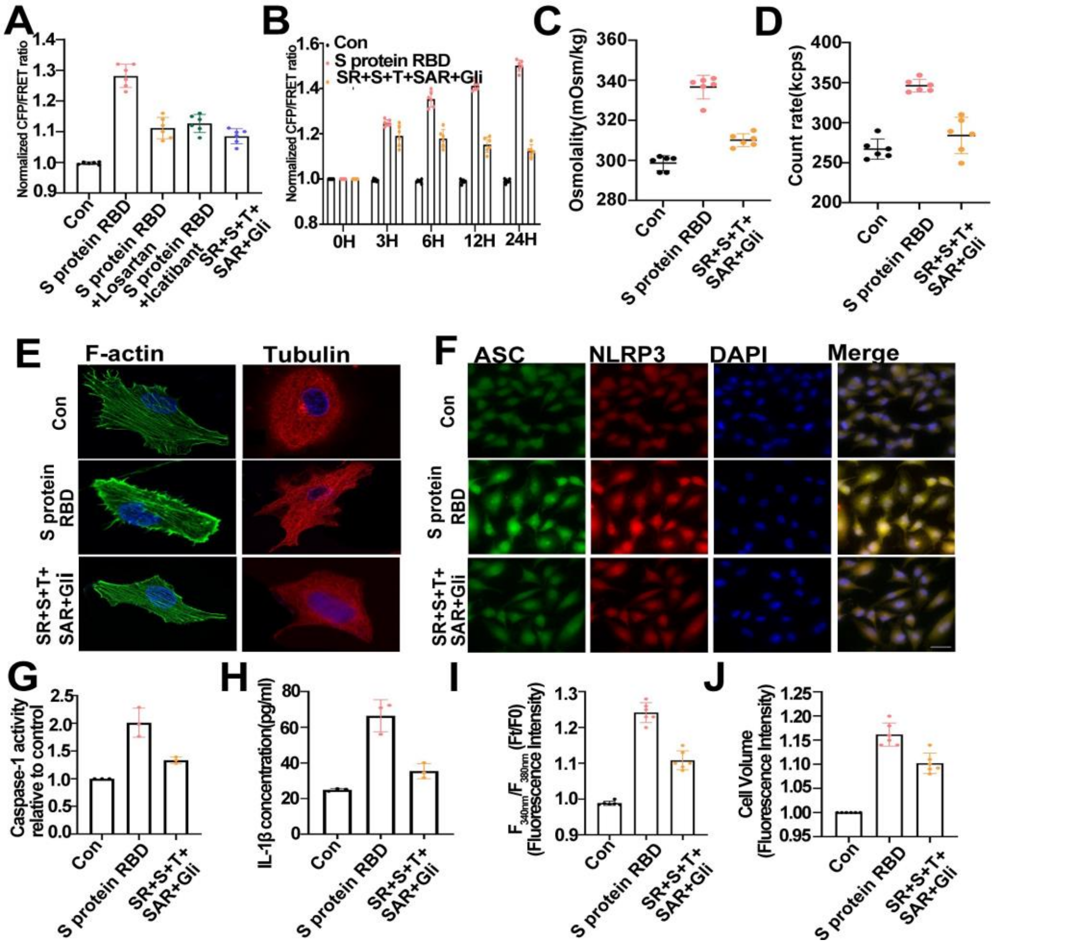

sFig.7 Changes in intracellular protein nanoparticle-related osmotic pressure and the potential roles of four selected drugs in response to the spike protein. (A) Normalization of CFP/FRET signals corresponding to vimentin-tension under spike protein, spike protein+Losartan, or spike protein+Icatibant stimulation at 15 min. (B) Normalized CFP/FRET ratios in vimentin of A549 cells were measured in response to Control, spike protein, and drug combinations at different times within 24 hours. (C and D) Cytoplasm osmotic pressure(left) and count rate of protein nanoparticle (Kcps (right) in A549 cells induced with Con, spike protein, and drug combinations, respectively, under isotonic conditions. (E) A549 cells stimulated with Con and spike protein were stained for Phalloidin(FITC) and α-tubulin (TRITC). Images were generated from confocal laser microscopy. (F) Immunofluorescence of ASC and NLRP3 in A549 cells. Scale bar: 20μm. (G and H) Cell lysate and the culture medium were collected and analyzed for Caspase-1 activity and IL-1β release, respectively. (I-J) Changes in the

fluorescence intensity of cytoplasmic calcium ions (I) and calcein-fluorescence intensity (J) in cells treated with Con, spike protein, and drug combinations. Average of ≥ 5 experiments ± SEM. ns, $p > 0.05$, **$p < 0.01$, ***$p < 0.001$.

Supplementary Fig. 8

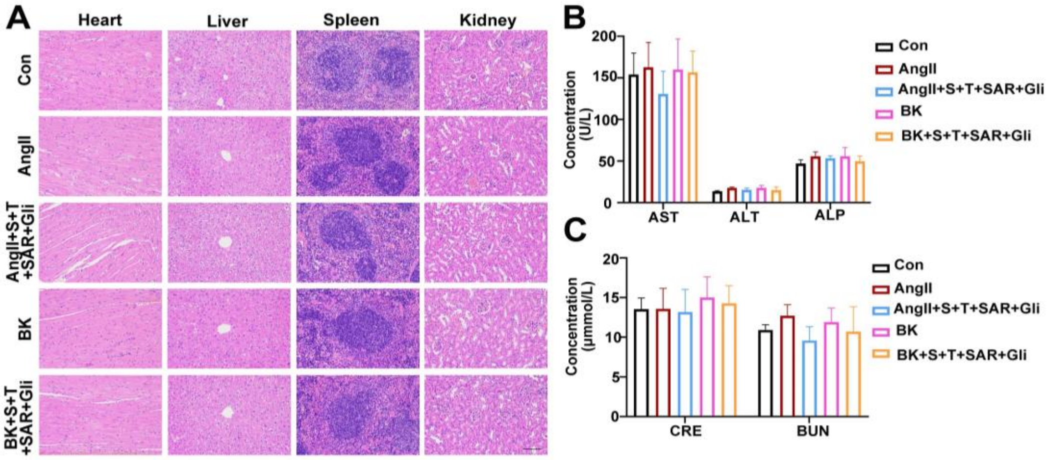

sFig.8 Safety assessment of the drug combination *in vivo*. (A) Representative images of H&E-stained histological sections of the major organs. Scale bar: 100μm. (B-C) Indicators reflected the blood biochemistry of the liver (B) and kidney (C) function. Average of ≥ 5 experiments ± SEM. ns, $p>0.05$, $^{**}p < 0.01$, $^{***}p < 0.001$.
